# An engineered periosteum for efficient delivery of rhBMP-2 and mesenchymal progenitor cells during bone regeneration

**DOI:** 10.1038/s41536-023-00330-2

**Published:** 2023-09-29

**Authors:** Juan Antonio Romero-Torrecilla, José María Lamo-Espinosa, Purificación Ripalda-Cemboráin, Tania López-Martínez, Gloria Abizanda, Luis Riera-Álvarez, Sergio Ruiz de Galarreta-Moriones, Asier López-Barberena, Naiara Rodríguez-Flórez, Reyes Elizalde, Vineetha Jayawarna, José Valdés-Fernández, Miguel Echanove-González de Anleo, Peter Childs, Elena de Juan-Pardo, Manuel Salmeron-Sanchez, Felipe Prósper, Emma Muiños-López, Froilán Granero-Moltó

**Affiliations:** 1 Cell Therapy Area, Clínica Universidad de Navarra, Pamplona, Spain; 2 Biomedical Engineering Program, Centro de Investigación Médica Aplicada (CIMA), Pamplona, Spain; 3 Department of Orthopedic Surgery and Traumatology, Clínica Universidad de Navarra, Pamplona, Spain; 4 Instituto de Investigaciones Sanitarias de Navarra (IdiSNA), Pamplona, Spain; 5 Tecnun-School of Engineering, Universidad de Navarra, San Sebastian, Spain; 6 IKERBASQUE, Basque Foundation for Science, Bilbao, Spain; 7 Center for the Cellular Microenvironment, James Watt School of Engineering, University of Glasgow, Glasgow, United Kingdom; 8 Department of Biomedical Engineering, University of Strathclyde, Glasgow, United Kingdom; 9 T3mPLATE, Harry Perkins Institute of Medical Research, Queen Elizabeth II Medical Centre and the UWA Centre for Medical Research, The University of Western Australia, Perth, Australia; 10 Centro de Investigación Biomédica en Red de Cáncer (CIBERONC), Pamplona, Spain; 11 Department of Hematology, Clínica Universidad de Navarra, Pamplona, Spain

**Keywords:** Regenerative medicine, Translational research, Biomaterials, Tissue engineering

## Abstract

During bone regeneration, the periosteum acts as a carrier for key regenerative cues, delivering osteochondroprogenitor cells and crucial growth factors to the injured bone. We developed a biocompatible, 3D polycaprolactone (PCL) melt electro-written membrane to act as a mimetic periosteum. Poly (ethyl acrylate) coating of the PCL membrane allowed functionalization, mediated by fibronectin and low dose recombinant human BMP-2 (rhBMP-2) (10-25 μg/ml), resulting in efficient, sustained osteoinduction in vitro. In vivo, rhBMP-2 functionalized mimetic periosteum demonstrated regenerative potential in the treatment of rat critical-size femoral defects with highly efficient healing and functional recovery (80%-93%). Mimetic periosteum has also proven to be efficient for cell delivery, as observed through the migration of transplanted periosteum-derived mesenchymal cells to the bone defect and their survival. Ultimately, mimetic periosteum demonstrated its ability to deliver key stem cells and morphogens to an injured site, exposing a therapeutic and translational potential in vivo when combined with unprecedentedly low rhBMP-2 doses.

## Introduction

The well recognized regenerative properties of bone tissue are not exempt from complications. High energy trauma or critical-size bone defects, derived from infections or tumors, will limit the reparative response. These complications and the affected patient’s comorbidities can result in delayed unions and even nonunion, with devastating consequences. Nonunion fractures are a major cause of chronic pain and disability and the cost of treating them is a significant burden for health care systems. Out of the millions of fractures reported globally, it is estimated that 5–10% will develop some healing disturbance or problem, including nonunion^1–3^.

Autologous bone graft is the gold standard for the treatment of large bone defects or nonunion fractures. Other surgical procedures in orthopaedics or dentistry require bone autograft to be effective^4^. In fact, bone is the second most grafted tissue in surgery, and it has been estimated that 2 million bone grafts are performed worldwide annually^5^. The major limitations of autografting are the amount of tissue available and the number of donor sites available. Although autografting is beneficial, it is not a risk-free procedure. Associated risks include pain and morbidity at the graft extraction site, infection, and elevated cost due to extended surgical times. In addition, the age and condition of patients could also limit the viability and efficacy of the bone graft. Allograft is a common alternative to autograft but has limited efficacy for critical-size defects and a propensity to fail in the long term^6^.

In orthopedic surgery, BMPs, and especially rhBMP-2, are becoming more widely recognized as being able to complement and even substitute grafting techniques^7^. BMP-2 is a growth factor that has a direct effect when committing progenitor mesenchymal cells to an osteoblastic lineage, both in vitro and in vivo^8^. Therapeutic rhBMP-2, and in general BMPs, need to be delivered locally. A wide variety of organic (bone demineralized matrix) or collagenous materials, gelatins, and inorganic materials (e.g., ceramics and titanium mesh) have been tested for this function^9–11^. Collagen presents interesting properties due to its biocompatibility, degradation, and cell interaction profile. Several clinical trials have evaluated the delivery of rhBMP-2 in combination with absorbable collagen sponges (ACS). To date, the Food and Drug Administration (FDA) and European Medicine Agency (EMA) have approved clinical treatment with rhBMP-2, based on ACS that is implanted at the fracture site and serves as a carrier of the morphogen. It also serves as a scaffold for new bone growth^12^. In orthopedics, the side effects associated with the use of rhBMP-2, namely ectopic bone formation, edema, bone resorption and even cancer, hamper the wider use of this osteogenic factor. It is believed that these side effects are a result of the off-label use of rhBMP-2 together with supraphysiological dosing^13–15^.

It is assumed that the need for supraphysiological dosing is due to the early burst release observed with administration of rhBMP-2 from collagen sponges^16^. To reduce the amount of rhBMP-2 delivered, different strategies of sustained release have been developed, including microparticle/microsphere encapsulation^17^, microparticle encapsulation embedded into scaffolds or hydrogels^18^, immobilization in functionalized scaffolds by affinity interactions with other proteins, through covalent binding^19,20^ or even viral vector transduction of mesenchymal stem cells^21^.

In vertebrates bone repair is directed by the periosteum, a vascularized membrane-like structure covering the bones containing skeletal progenitor cells that express and secrete BMP-2 in response to bone tissue injury^22,23^. In humans, the periosteum represents two structurally and functionally differentiated zones. The cambium layer, i.e., the inner part in contact with the bone tissue which contains skeletal progenitor cells, and the fibrous layer, which has less cellular content and allows muscle attachment^24,25^. In situations where bone trauma damages the periosteum, or limits the expression of *BMP2* by mesenchymal progenitors, fracture healing may be delayed or ultimately result in nonunion^26,27^. Periosteal autografting has been used as a substitute for bone autografting in experimental models of bone regeneration. Whether it is used to complement allograft, or on its own, periosteal grafting has been reported as improving bone integration and repair^28,29^. Nevertheless, the availability of healthy periosteum is also a limiting factor that restricts its clinical use. As a result, over the last few years, various groups have become increasingly aware of the advantages related to bioengineering a periosteum-like structure. Initially, decellularized periosteum was employed as a substrate to further integrate skeletal stem cells but met with low success rates^30^. Another interesting approach was to use a periosteum-like membrane as a scaffold to further fill up the defect with synthetic bone (tricalcium phosphate) or biocompatible polymers^31^. The use of graft based on gelatin membranes, mimicking the periosteum to host cells and vessels, has also been thoroughly studied^32^. These structures have demonstrated their capacity to bind and deliver various osteoinductive factors, as well as facilitate vasculature invasion. Nevertheless, a failure to fully characterize the delivery of these factors has led to incomplete therapies, showing poor healing response.

In this work we fabricated a 3D engineered periosteum using melt electro-written poly ε-caprolactone (PCL). We evaluated the therapeutic potential of the synthetic periosteum using different approaches for functionalization. These strategies include vascularization by an induced membrane-like approach^33^, low concentration rhBMP-2 binding^34^, or low concentration rhBMP-2 supplemented with periosteum derived mesenchymal progenitor cells (PMSCs). We assessed their regenerative properties in vivo using a critical size defect of the femur in immunocompetent rats.

## Results

### Design, fabrication, and assembly of a mimetic periosteum

**Fig. 1:**
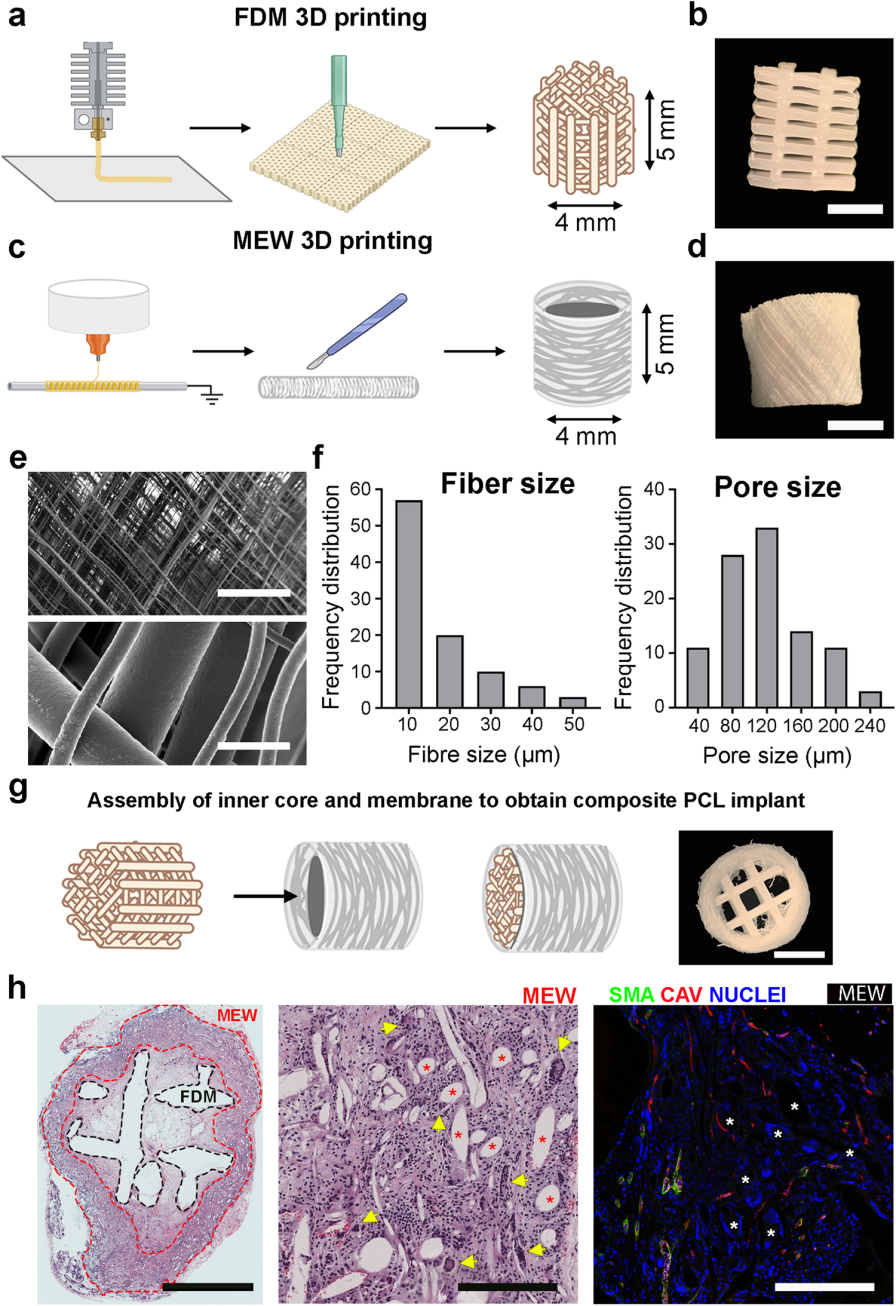
Design, fabrication, and characterization of a bone implant containing a mimetic periosteum that promotes vascularization. **a** Schematic of the 3D printing scaffold by FDM (fused deposition modeling) and extraction of the inner component of the implant. **b** Bright field image of the final inner scaffold after extraction; scale bar = 2 mm. **c** Schematic of the 3D printed scaffold by MEW (melt electrowriting), final dimensions of the mimetic periosteum are 4 mm diameter, 5 mm height. **d** Bright field picture of the final 3D MEW scaffold/mimetic periosteum; scale bar = 2 mm. **e** SEM (scanner electron microscope) image of the 3D MEW scaffold showing the porous nature of the mimetic periosteum and fiber size; scale bar upper image = 500 µm; scale bar lower image = 25 µm. **f** Fiber size frequency distribution of the MEW membrane graphic in microns. Pore size frequency distribution graph expressed in microns for the MEW mimetic periosteum. **g** Schematic of inner implant core and MEW membrane assembly and bright field image of the whole implant, scale bar = 2 mm. **h** Histological evaluation of the implants after 6 weeks of ectopic implantation. Left image shows a coronal section stained with H&E of the whole implant; scale bar = 2 mm. Middle image shows a magnification of the previous H&E staining, red asterisks mark position of MEW fibers, yellow arrow heads point giant multinucleated cells associated with these MEW fibers; scale bar = 200 µm. Right image shows double immunostaining (Cav Caveolin, SMA αSMA) for vessel detection in response to foreign body reaction, white asterisks mark for MEW fibers; scale bar = 200 µm.

To supplement bone regeneration strategies, we designed an implant where functional and active treatments would be applied from a scaffold mimicking the periosteum. Additive manufacturing has demonstrated the ability to obtain 3D scaffolds with well-defined properties and tunable architectures^35,36^. Therefore, the implant was designed as the combination of an inner and an outer 3D PCL scaffold with functional and structural differences. To provide mechanical stability, the inner scaffold was printed by fused deposition modeling (FDM) as square sheets (100 mm side, 5 mm thickness) presenting a pore size of 1.5 mm wide and 0.5 mm high (Fig. 1a). The final cylindrical scaffold, which measures 4 mm in diameter and 5 mm in height, was extracted by manually using a 4 mm diameter biopsy punch (Fig. 1b). The outer scaffold, forming the mimetic periosteum membrane, was printed using the melt electro-writing (MEW) technique resulting in a 70 mm tube, which was later cut down into 5 mm long pieces 4 mm in diameter (Fig. 1c, d). To determine microscale structure and spatial distribution of the MEW mimetic periosteum membrane, samples were subjected to scanning electron microscopy (SEM). SEM showed that a growing number of pores were distinguishable as magnification increased. At maximum magnification, individual fibers with sizes from 10 µm to 50 µm in diameter were visible. The distance between fibers indicated an apparent pore size between 40 and 240 µm, with 120 µm being the most frequent size (Fig. 1e, f). The final implants were assembled by combining both PCL structures (Fig. 1g).

To determine if the porous structure is compatible with vascularization and cell invasion, we ectopically implanted assembled scaffolds into the back of Sprague-Dawley (SD) rats for 6 weeks and evaluated the presence of vascularization. Histological staining with H&E allowed differentiation of the FDM scaffold and MEW membrane, with both scaffolds presenting cellular invasion (Fig. 1h, left panel). Magnification of the outer fibrillar MEW scaffold showed high cellularity associated with abundance of multinucleated giant cells (MNGCs) (Fig. 1h, central panel, arrow heads) suggesting a foreign-body reaction (FBR) against MEW PCL fibers^37^. Anti-CD68 immunostaining intensely labeled the MEW outer scaffold, allowing us to corroborate that these multinucleated cells were macrophage-derived giant cells (Supplemental Fig. 1 a–d, arrows). Immunohistological analysis showed that cellular infiltration included the presence of vascular invasion between the PCL fibers (caveolin, αSMA positive cells)^27,38–41^ (Fig. 1h, right panel).

We conclude that the designed porosity of the mimetic periosteum is competent at allowing vascularization.

### Ectopic implantation promotes increased vascularization and induction of Masquelet-like induced membrane

To determine the in vivo effects of the mimetic periosteum, we performed orthotopic implantation of the non-functionalized implant into a critical-size femoral defect of SD rats for 10 weeks^42^. After this period, rat thighbones were extracted and subjected to H&E staining to assess the histology of the implants. From these tissue samples we were able to identify the inner FDM scaffold surrounded by fibrillar MEW membrane. Histological magnification showed that orthotopic implantation also results in a foreign body reaction and presence of MNGCs that were reactive for an antibody against CD68 (Supplementary Fig. 1e–h).

**Fig. 2:**
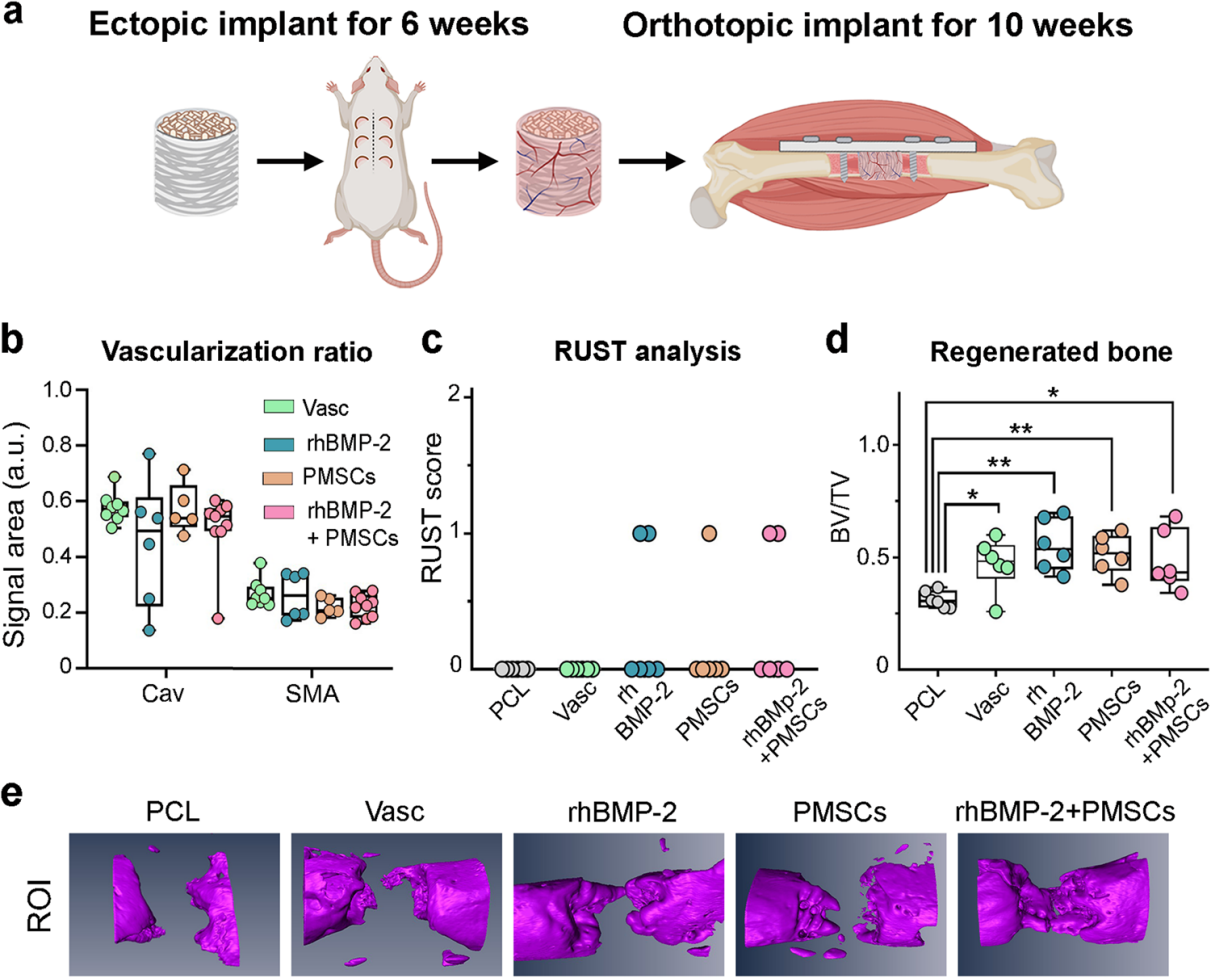
Mimetic periosteum promotes formation of a Masquelet’s induced-like membrane. **a** Experimental design for functionalization, vascularization of the mimetic periosteum. **b** Quantification of the vascular invasion in response to the implanted treatments 6 weeks ectopic subcutaneous implantation. Vasc, *n* = 8; rhBMP-2, *n* = 6; PMSCs, *n* = 5; rhBMP-2 + PMSCs, *n* = 9. Cav Caveolin, SMA αSMA. Results expressed as a median with an interquartile range, whiskers representing minimum and maximum values. **c** Simplified radiographic scoring RUST and healing after orthotopic implantation of the vascularized implants. **d** Quantification of bone regeneration at the site of implantation (*n* = 6 per group). PCL non-vascularized implant, *n* = 6; VASC ectopically vascularized implant without treatment, *n* = 6; rhBMP-2 ectopically vascularized implant with 1 µg of rhBMP-2, *n* = 6; PMSCs, ectopically vascularized implant with 3E6 rPMSCs, *n* = 6; rhBMP-2 + PMSCs, ectopically vascularized implant with rhBMP-2 (1 µg) and rPMSCs (3E6 rPMSCs), *n* = 6. Significance was calculated by one way ANOVA (*F* = 4.720, *p* = 0.0056) followed by Dunnett’s multiple comparisons test. *p = 0.0486 (PCL *vs* VASC), *p* = 0.0254 (PCL *vs* rhBMP-2 + PMSCs); **p = 0.0017 (PCL *vs* rhBMP-2), *p* = 0.0090 (PCL *vs* PMSCs). Results expressed as a median with an interquartile range, whiskers representing minimum and maximum values. **e** 3D reconstruction of new bone formation at the region of interest (ROI).

These results suggest that our mimetic periosteum design allows the formation of an induced membrane when orthotopically or ectopically implanted. We therefore sought to functionalize the synthetic periosteum by mimicking Masquelet’s technique for induced membrane. This is a two-stage surgical procedure proven to be clinically successful in the treatment of large bone defects^43^. For the first step, full constructs were implanted subcutaneously for 6 weeks to trigger the formation of the induced membrane, which was then followed by a second step where vascularized constructs were extracted from the donor animal and immediately orthotopically implanted into a critical-size femoral defect for 10 weeks (Fig. 2a). Double immunostaining against caveolin and αSMA was performed to assess vascular abundance and maturity (Fig. 2b). Interestingly, adding rhBMP-2 (1 μg per implant), rPMSCs (3 million cells per implant) or a mix of rPMSCs and rhBMP-2 (3 million cells, 1 μg) has negligible impact on the generation of vascularization ectopically, as determined by caveolin or αSMA quantification (Fig. 2b). The osteogenic effect of ectopically vascularized mimetic periosteum was also assessed orthotopically in our critical size defect model of the femur. All the induced membrane groups generated significantly more new bone formation than non-functionalized controls. However, none of the animals presented complete healing or cortical continuity when assessed by X-ray radiography or micro computed tomography (μCT) (Fig. 2c–e).

The high porosity, biocompatibility, and structure of the implants demonstrated a major ability to undergo vascular invasion, even in the absence of further treatment, but further functionalization would be needed for therapeutic use.

### Polymeric PEA functionalization allows for rhBMP-2 dose standardization and promotes high osteogenic and osteoinductive potential in vitro

**Fig. 3:**
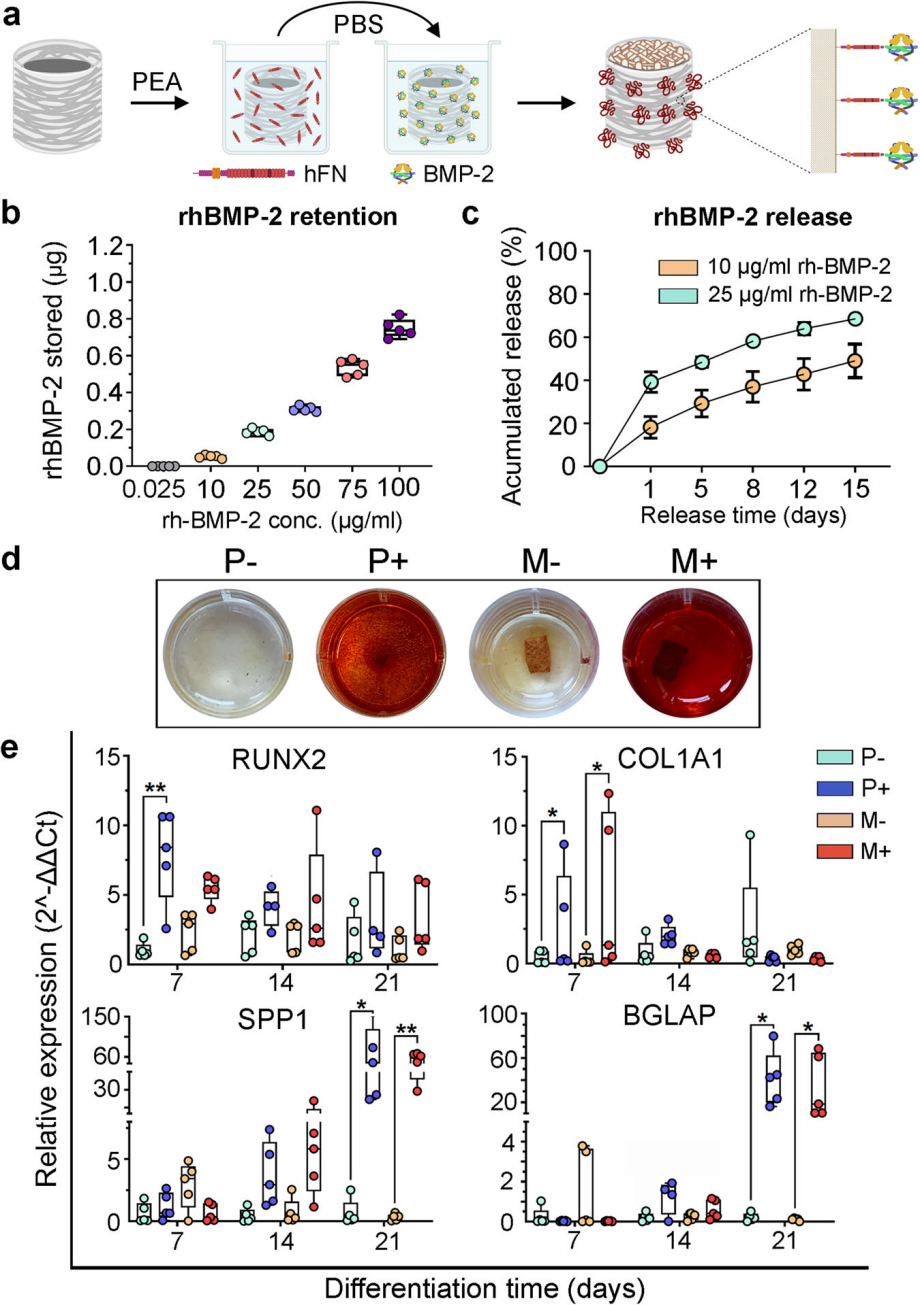
Functionalization with rhBMP-2 of the mimetic periosteum and evaluation of the osteoinductive properties in vitro. **a** Strategy for mimetic periosteum functionalization mediated by PEA and fibronectin coating. **b** Quantification of the rhBMP-2 bound to the mimetic periosteum after incubation with grading concentrations of rhBMP-2. Results expressed as a median with an interquartile range, whiskers representing minimum and maximum values. **c** Kinetic release of rhBMP-2 from selected membranes for 15 days. Error expressed as SD. **d** Alizarin Red staining of osteoblast differentiation of hPMSCs induced with rhBMP-2. P− cell monolayer in culture plate, P+ cell monolayer in culture plate + rhBMP-2, M− cells in membrane, M+ cells in membrane + rhBMP-2. **e** Transcriptional response to rhBMP-2 during different stages of the osteogenic differentiation, early (*RUNX2*, *COL1A1*) and late (*SPP1*, *BGLAP*) osteoblast markers, *n* = 5 independent human donors. Significance was determined by Kruskal–Wallis test followed by Dunnett’s multiple comparisons test. *p < 0.05; **p < 0.01. *RUNX2*, *p* = 0.0066 (P− vs P+, 7 d); *COL1A1*, *p* = 0.0286 (P− *vs* P+ and M− *vs* M+); *SPP1*, *p* = 0.0108 (P− *vs* P+) and *p* = 0.0055 (M− *vs* M+); *BGLAP*, *p* = 0.0176 (P− *vs* P+) and *p* = 0.0128 (M− *vs* M+). Results expressed as a median with an interquartile range, whiskers representing minimum and maximum values.

To further determine the therapeutic potential of the MEW membranes, we designed a strategy for functionalization with rhBMP-2. For this purpose, we performed radical polymerization of poly (ethyl acrylate) coating (PEA) onto the outer mimetic periosteum surface, followed by human fibronectin (hFN) and rhBMP-2 binding^34,44,45^ (Fig. 3a). Interestingly, we found that increasing rhBMP-2 incubation concentration linearly correlates with the dose of rhBMP-2 stored in the mimetic periosteum (Fig. 3b). Kinetic release from implants incubated in 10 and 25 µg/ml (net dose of 55 ng and 190 ng respectively) of rhBMP-2 share a similar profile throughout the 15-day period of the release study, although the 10 µg/ml sample releases a lower percentage of the respective stored dose (Fig. 3c).

To determine the osteoinductive and osteogenic potential of the functionalized mimetic periosteum, human periosteum-derived mesenchymal progenitor cells (hPMSCs) underwent osteogenic differentiation on the surface of the MEW membrane for 21 days without (M−) or with functionalization (M+) with rhBMP-2 (25 μg/ml, 190 ng). By way of performing a positive control of the differentiation process, hPMSCs were differentiated on culture plates in the absence (P−) or the presence (P+) of rhBMP-2 (200 ng) in the differentiation medium. Osteogenic differentiation was assessed by Alizarin Red staining. Only cultures receiving rhBMP-2, either when grown on the surface of the cell culture plate (P+) or the membrane (M+), were positive for the staining (Fig. 3d). The osteogenic differentiation was also assessed at the transcription level by the relative expression of key genes for early and mature osteoblast differentiation (*RUNX2, COL1A1, BGLAP, SPP1*). On the one hand, *RUNX2* and *COL1A1* expression were significantly higher for hPMSCs differentiated on plate or membrane when compared to the negative controls (P−, M−). This occurred at 7 days post differentiation induction, which suggests an early commitment to the osteoblastic lineage. On the other hand, commitment into advanced or mature osteoblasts was demonstrated by a significantly higher expression of osteopontin (*SPP1*) and osteocalcin (*BGLAP*) after induction for 21 days in both modalities of rhBMP-2 treatment (Fig. 3e). It is important to note that there are no major transcriptional differences between hPMSCs differentiated on membrane substrates and those differentiated in plates. However, the M+ group underwent the whole differentiation process with the initial 190 ng of rhBMP-2 delivered from the membrane, whilst in the P+ group all media changes (performed every 2-3 days) included 200 ng of fresh rhBMP-2.

These cellular and transcriptional results suggested that the engineered mimetic periosteum functionalized with rhBMP-2 had osteoinductive and osteogenic properties and was able to induce commitment of hPMSCs into mature osteoblasts with only an initial 190 ng of rhBMP-2 loaded on its surface.

### PEA-hFN-rhBMP-2 functionalized mimetic periosteum promotes highly effective critical-size femoral defect healing in vivo

**Fig. 4:**
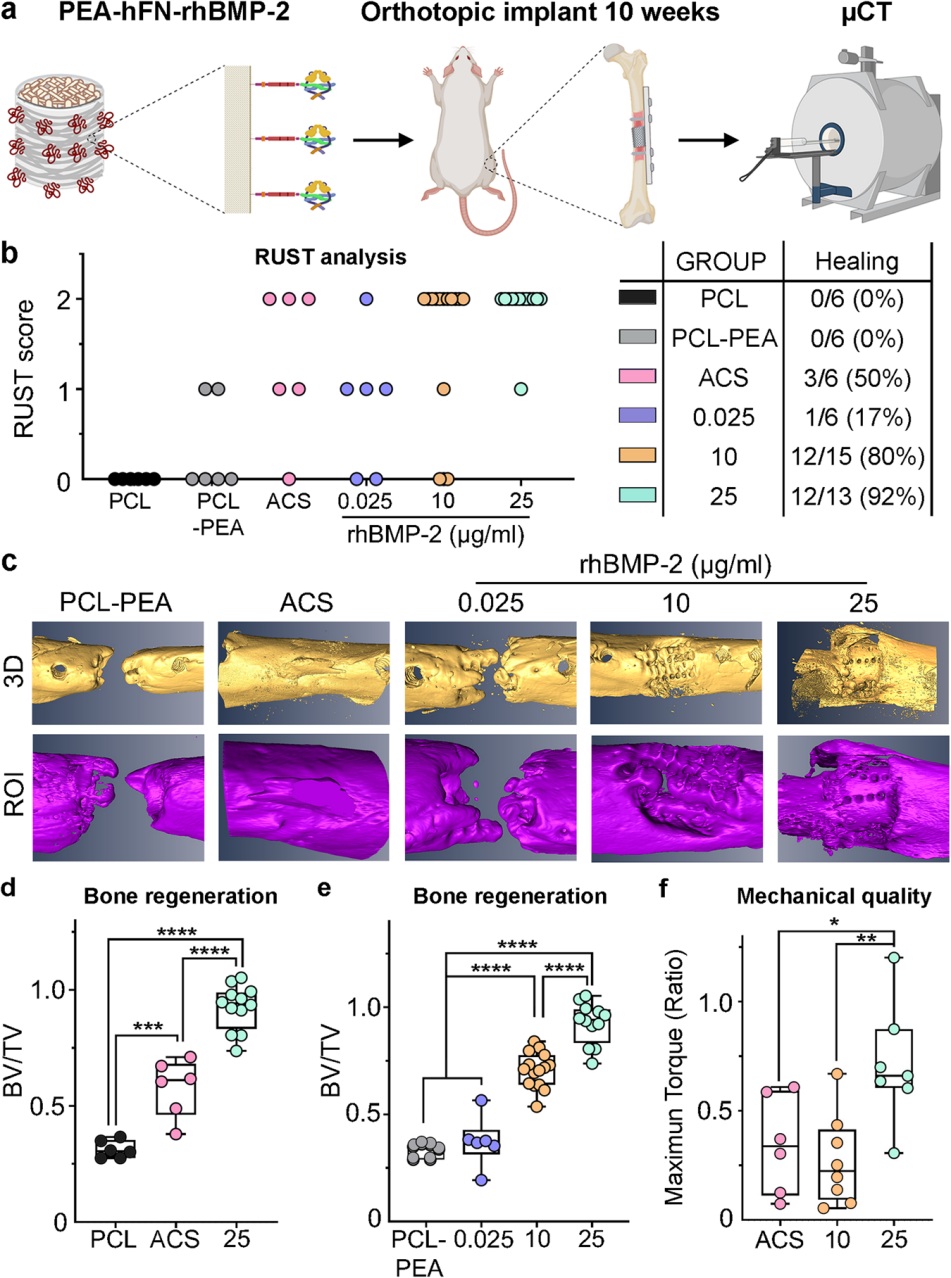
Efficient bone regeneration through functionalization with PEA-hFN-rhBMP-2 of the mimetic periosteum. **a** Graphical representation of the experimental design for in vivo evaluation of mimetic periosteum functionalization. **b** Radiographic analysis of the healing process (RUST) and efficiency of healing. PCL mimetic periosteum implant without treatment, PCL-PEA mimetic periosteum implant with outer membrane treated with PEA, ACS rhBMP-2 (190 ng) delivered from absorbable collagen sponge, 0.025 mimetic periosteum functionalized with PEA-FN and rhBMP-2 at 0.025 µg/ml, 10 mimetic periosteum functionalized with PEA-FN and rhBMP-2 at 10 µg/ml corresponding to 55 ng net dose, 25 mimetic periosteum functionalized with PEA-FN and rhBMP-2 at 25 µg/ml corresponding to 190 ng net dose. **c** Three dimensional renderings of the new bone formed at the defect site after 10 weeks of orthotopic implantation. **d** Quantification of the ratio of new bone formed at the implantation site (BV/TV). Left panel, bone regeneration of the mimetic periosteum compared with the standard ACS treatment with equivalent rhBMP-2 content (190 ng). PCL non-functionalized scaffold, *n* = 6; ACS absorbable collagen sponge, *n* = 6; 25 PCL scaffold functionalized with 25 μg/ml of rhBMP-2, *n* = 13. Significance was determined by one way ANOVA (*F* = 89.39, *p* < 0.0001) and Tukey’s multiple comparisons test. ***p < 0.001 (*p* = 0.0002, PCL *vs* ACS); ****p < 0.0001 (PCL *vs* 25 & ACS *vs* 25). Results expressed as a median with an interquartile range, whiskers representing minimum and maximum values. **e** Bone regeneration for mimetic periosteum implants functionalized with increasing concentrations of rhBMP-2. PCL-PEA PCL scaffold functionalized with fibronectin (Fn), *n* = 8; 0.025 PCL scaffold functionalized with Fn and 0.025 μg/ml of rhBMP-2, *n* = 8; 10 PCL scaffold functionalized with Fn and 10 μg/ml of rhBMP-2, *n* = 15; PCL scaffold functionalized with Fn and 25 μg/ml of rhBMP-2, *n* = 13. Significance was determined by one way ANOVA (*F* = 82.12, *p* < 0.0001) and Tukey’s multiple comparisons test. ****p < 0.0001. Results expressed as a median with an interquartile range, whiskers representing minimum and maximum values. **f** Mechanical testing of the bones repaired with rhBMP-2, maximum torque of the operated femurs is normalized with the maximum torque of the contralateral femur and expressed as a ratio (ACS, *n* = 6; 10, *n* = 8; 25, *n* = 7). Significance was determined by one way ANOVA (*F* = 7.149, *p* = 0.0052) followed by Tukey’s multiple comparisons test. *p = 0.0307; **p = 0.0055. Results expressed as a median with an interquartile range, whiskers representing minimum and maximum values.

To determine whether the mimetic periosteum had a therapeutic effect in vivo, implants loaded with different low rhBMP-2 dosages were tested in SD rats with a femoral critical-size defect model for 10 weeks and analyzed radiographically by μCT (Fig. 4a). With regards to the regeneration level, as determined by RUST scoring (detailed explanation of RUST scoring in Supplemental Fig. 2), all groups showed some level of regeneration except for the untreated implants (PCL) (Fig. 4b). Three-dimensional reconstruction of bone tissue by µCT showed that the positive control group, i.e., absorbable collagen sponge (ACS) with 190 ng of rhBMP-2, presented a large amount of regenerated bone, although only half the animals presented cortical continuity or full healing (Fig. 4b, c). It is noteworthy that the engineered mimetic periosteum coated with PEA-hFN-rhBMP-2 (PCL-PEA) and treated with an equivalent amount of rhBMP-2 (25 μg/ml, bulk 190 ng dose), also presented high levels of newly formed bony tissue by µCT with full healing and cortical continuity (12/13, 92%) (Fig. 4b, c). In addition, when the functionalized implants were loaded with a reduced dose of the morphogen (10 μg/ml, bulk 55 ng dose), high levels of bone regeneration could still be observed by μCT quantification and 3D reconstruction. In this case up to 12/15 animals presented full healing with cortical continuity, which represents an efficacy of 80% (Fig. 4b, c). It is worth mentioning that the ultralow dose group (0.025 μg/ml, below detection by ELISA) did not show healing efficiency but still managed to fully heal 1/6 animals (17%).

In terms of total content of rhBMP-2, the ratio of bone volume generated (BV/TV) by the PEA-FN-rhBMP-2 MEW membranes (25 μg/ml, 190 ng) was significantly higher than in the untreated group (PCL) as well as the standard treatment group using collagen sponge loaded with an equivalent amount of rhBMP-2 (ACS, 190 ng) (Fig. 4d). When we analyzed the ratio of bone volume generated (BV/TV) by PEA-FN-rhBMP-2 MEW membranes laden with different rhBMP-2 doses, we obtained a significantly higher ratio presented by the 190 ng (25, 25 μg/ml) dose compared to the 55 ng dose (10, 10 μg/ml) (Fig. 4e).

The bone quality after different treatments with rhBMP-2 was also evaluated by biomechanical testing. Biomechanical testing was carried out by a torsion test of the surgically treated femurs, using the contralateral femurs as controls. The maximum torque of all repaired bones was reduced when compared with the contralateral, intact bones. However, these differences were only significant when rhBMP-2 was applied from ACS (190 ng) or with the mimetic periosteum with the lowest concentration (10 μg/ml, 55 ng) (Supplementary Fig. 3). In fact, when normalized (Max torque repaired/Max torque contralateral), the maximum torque of the mimetic periosteum loaded with 25 μg/ml of rhBMP-2 showed significant differences when compared with both ACS and mimetic periosteum loaded with 10 μg/ml. Notably, the mechanical properties of the bones treated with the mimetic periosteum loaded with 10 μg/ml, 55 ng, were comparable to the ACS loaded with 190 ng of rhBMP-2 (Fig. 4f).

Finally, given the elevated number of animals that underwent full healing (80%), we undertook further assessment of the therapeutic potential of PEA-FN-rhBMP-2 treated MEW membrane with a low dose of morphogen (10 μg/ml, 55 ng). In this regard, we performed in vitro experiments with this membrane containing 55 ng rhBMP-2 but added a lyophilization treatment. Lyophilized mimetic periosteum showed a high rate of healing, with 5/6 animals presenting regenerated femur (Supplementary Fig. 4a), while there were negligible differences between a lyophilized membrane and a freshly prepared one together with a high amount of freshly formed bone, as indicated by bone volume/tissue volume ratio (BV/TV) and 3D reconstruction (Supplementary Fig. 4b, c).

This data suggests that lyophilization would allow for a ready-to-use membrane in future clinical treatments.

### Functionalized PCL mimetic periosteum drives bone growth

**Fig. 5:**
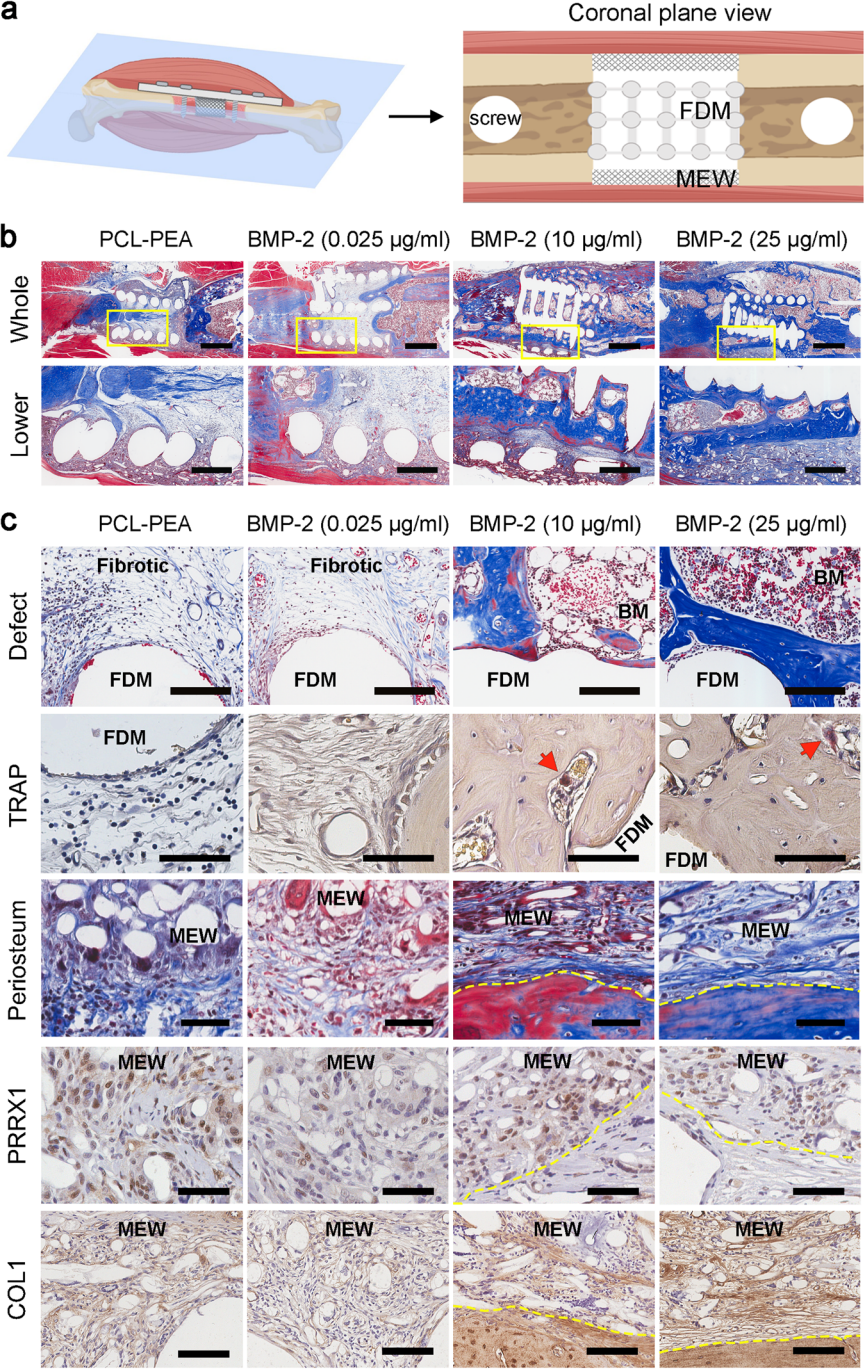
Histological assessment of bone regeneration mediated by the mimetic periosteum functionalized with rhBMP-2. **a** Schematic interpretation of the histological samples processing. **b** Masson’s trichrome staining of the groups functionalized with PCL-PEA-hFN-rhBMP-2 with different doses and control without rhBMP-2 (PCL-PEA). Upper row (Whole), general view of the ROI, scale bar = 2 mm. Lower row, detail of the boundaries between outer mimetic periosteum and inner FDM scaffold; scale bar = 500 µm. **c** Histological and immunohistochemical assessment of the biological quality of regenerated bone tissue. Upper row shows a magnification of the defect area for all groups (BM = bone marrow) stained with Mason´s Trichrome (MT); scale bar = 150 µm. Second row, TRAP staining for the injury area around the inner FDM scaffold, red arrow heads pointing osteoclasts in successful therapies; scale bar = 75 µm. Third row shows MT stained newly formed periosteum marked in yellow dotted line within MEW fibers; scale bar = 100 µm. Fourth and fifth rows display magnification of IHC for this area (PRRX1, anti-PRRX1 and COL1, anti-type I collagen). Yellow dotted lines mark the boundaries of the new formed periosteum at the MEW scaffold; scale bar = 75 µm.

The quality of the bone regeneration mediated by the mimetic periosteum, functionalized with rhBMP-2, was also assessed histologically. We positioned the femur samples for sectioning and viewed the coronal plane of the images. Arrow heads on the right panel indicate how we expected to visualize holes left by screws, FDM-PCL inner scaffold, and MEW membrane meshes from left to right respectively (Fig. 5a). Masson’s trichrome staining showed little new bone formation and growth in the defect in both the control group (PCL-PEA) and in mimetic periosteum with ultralow rhBMP-2. However, groups in which the mimetic periosteum was functionalized with low concentrations of rhBMP-2 (10 µg/ml, 25 µg/ml) showed abundant newly formed bone tissue (Fig. 5b, upper row, Supplementary Figs. 5 to 8). Higher magnification of the defect area showed bone growth confined inside of the mimetic periosteum in all groups (Fig. 5b, lower row).

Histological analysis showed that regenerated bone from 10 and 25 μg/ml functionalized scaffolds result in good quality bone at 10 weeks post-surgery, characterized by the presence of consolidated bone marrow within the core. Additionally, TRAP analysis showed low presence of osteoclasts, with similar levels to normal trabecular bone (Fig. 5c, Supplementary Fig. 9). We detected that the formation of new bone in the defect core is associated with the presence of a periosteum-like tissue that forms at the MEW scaffold (Fig. 5c, yellow dot lines). Immunohistological analysis for PRRX1, a mesenchymal progenitor marker, and type I collagen, a major component of the periosteum ECM, showed abundant positive signal in the invading cells and fibers of the MEW scaffold (Fig. 5c).

These data suggest that low rhBMP-2 dosing, delivered from the mimetic periosteum, produces a good quality bone tissue and the reconstitution of a native periosteum, preventing ectopic bone formation.

### Mimetic periosteum acts to deliver rPMSCs with therapeutic effects, but they are not able to integrate in the long term

We have previously demonstrated that PMSCs are a suitable MSC population for cell therapies directed at bone regeneration. When applied in critical-size defects in rats rPMSCs demonstrated better survival and better regenerative outcomes than bone marrow derived MSCs. However, no integration was detected after 10 weeks of implantation. We and others have demonstrated that the regenerative improvements observed with the use of MSCs could be explained by trophic factors, including BMP-2, secreted by transplanted cells during the early weeks of MSCs presence^46–48^.

To mimic the native periosteum function, and increase MSC survival and integration, we assessed the ability of our implant to deliver rhBMP-2 together with periosteum derived MSCs (PMSCs).

Rat periosteum derived MSCs (rPMSCs) were isolated from the periosteum of hindlimb bones of transgenic SD rats that express constitutively EGFP, allowing further tracking of the fate of donor cells. In addition, mimetic periosteum was previously functionalized with rhBMP-2 (10 μg/ml).

**Fig. 6:**
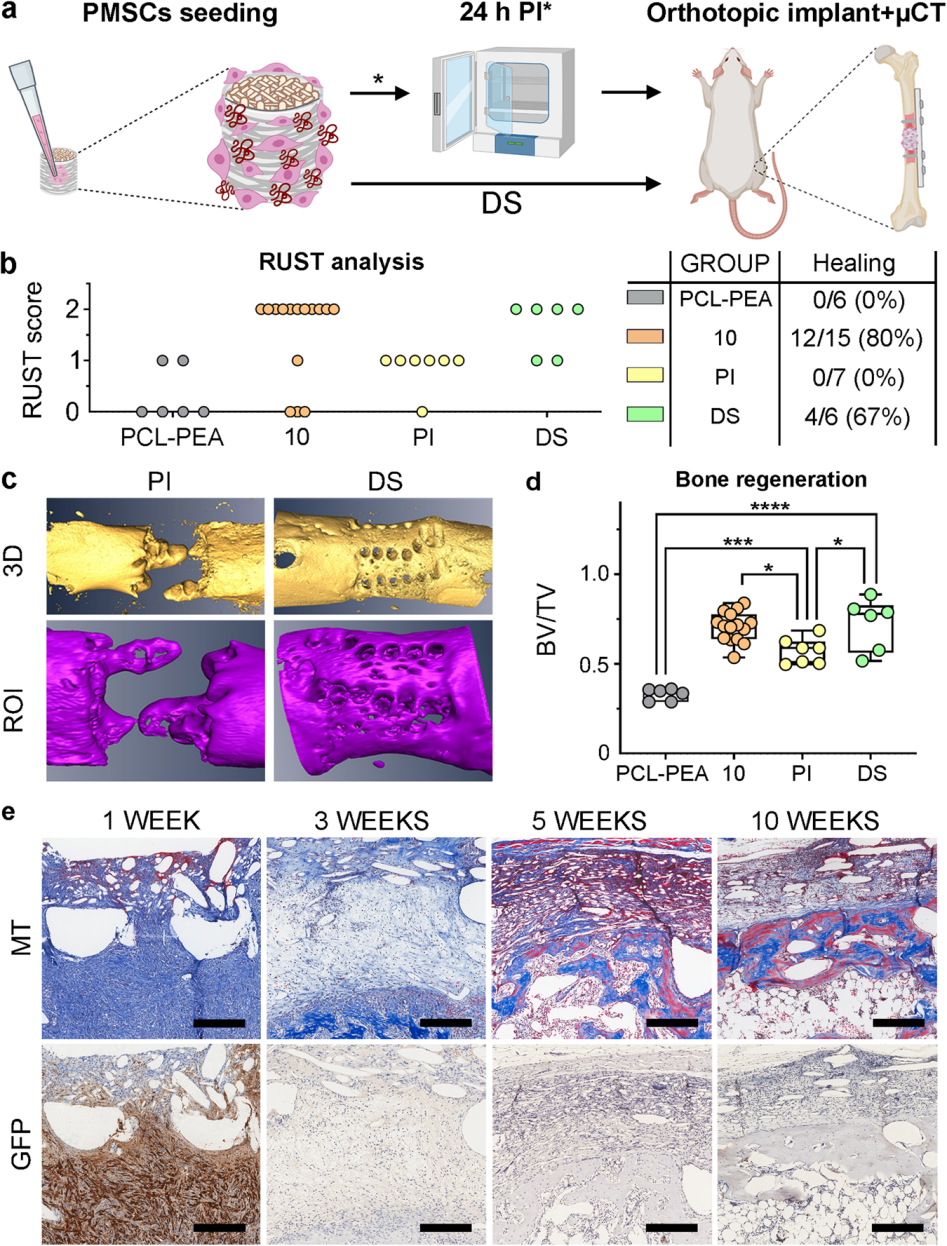
Cellular functionalization of the mimetic periosteum. **a** Schematic of the experimental design for functionalization with rPMSCs. PI pre-incubation for 24 h in vitro, DS direct seeding before implantation. **b** Radiographic scoring (RUST) and healing evaluation (PCL-PEA, non-treated implant; 10, mimetic periosteum functionalized with PEA-FN and rhBMP-2 at 10 µg/ml corresponding to 55 ng net dose; PI mimetic periosteum functionalized with PEA-FN and rhBMP-2 at 10 µg/ml corresponding to 55 ng net dose and 3E6 rPMSCs pre-incubated 24 h; DS, mimetic periosteum functionalized with PEA-FN and rhBMP-2 at 10 µg/ml corresponding to 55 ng net dose and 3E6 rPMSCs directly seeded into the mimetic periosteum and implanted). **c** Three-dimensional renderings of the new bone formation at the implant site (ROI). **d** Quantification of the new formed bone at the ROI. PCL-PEA, untreated implant (*n* = 6); 10, mimetic periosteum functionalized with 10 µg/ml of rhBMP-2 (*n* = 15); PI, mimetic periosteum functionalized with 10 µg/ml of rhBMP-2 and 3E6 rPMSCs (*n* = 7); DS, mimetic periosteum functionalized with 10 µg/ml of rhBMP-2 and 3E6 rPMSCs (*n* = 6). Significance was determined by one way ANOVA (*F* = 31.39, *p* < 0.0001) and Tukey’s multiple comparisons test. *p = 0.0203 (DS *vs* PI), *p* = 0.0127 (10 *vs* PI); ***p = 0.0002 (PCL-PEA *vs* PI); ****p < 0.0001 (PCL-PEA *vs* DS). Results expressed as a median with an interquartile range, whiskers representing minimum and maximum values. **e** Histological assessment (MT, Masson’s trichrome staining) and tracking by IHC (GFP, anti-GFP) of the transplanted rPMSCs along the reparative process. MEW melt electro-written scaffold, FDM fused deposition modeling printed scaffold. Scale bar = 250 µm.

We assessed this with two different strategies: 1) seeding rat PMSCs into the mimetic periosteum 24 h before orthotopic implantation (PI); and 2) direct cellular seeding in the surgical room right before implantation (DS) (Fig. 6a).

After 10 weeks, radiographic analysis (RUST) indicated that the PI treated group showed no animals to have healed, whereas the direct rPMSCs-seeded group had a total of 67% healed, which indicates that preincubation of cells and implant drastically diminishes the osteogenic and osteoinductive potential (Fig. 6b). Radiographic evaluation was confirmed after 3D reconstruction and quantification of newly formed bone. The PI group showed negligible newly formed tissue while the DS group showed robust regenerated tissue and cortical continuity in most of the animals treated (Fig. 6c). Quantification of bone production, expressed as BV/TV, indicates that both cellular treatments result in a significant increase in bone volume when compared to non-functionalized implants. However, none of these achieved a significant therapy improvement when compared to implants functionalized only with rhBMP-2 treatment (10 μg/ml) (Fig. 6d). Finally, EGFP-rPMSCs seeded directly without preincubation were tracked at different stages of the reparative period (1-, 3-, 5- and 10-weeks post-surgery) by IHC using anti-GFP immunostaining. It is worth highlighting that cells seeded in the mimetic periosteum were later found at the inner scaffold, indicating that active migration of the cells from the mimetic periosteum occurred at least until 1-week post fracture. Nevertheless, we were unable to detect GFP signal after three weeks post-fracture, even in the vicinity of the mimetic periosteum or the areas of osteogenic activity (Fig. 6e).

Importantly, our mimetic periosteum represents an optimal platform for delivery of both rhBMP-2 and progenitor cells.

## Discussion

Autograft is considered the gold standard for treating bone defects, high energy fractures, and nonunions. However, the availability of such grafts is limited, especially in the treatment of large bone defects^49^. In the last few years, tissue engineering has become an attractive field, providing new approaches for treating different diseases, and successfully combining materials and biological factors^50–53^. It has been extensively proven that periosteum (a vascularized fibrous connective tissue covering the outer surface of bone), plays a key role in bone regeneration by releasing not only mesenchymal stem cells, but also key growth factors, such as BMP-2 and other osteogenic metabolites^22–24,54,55^.

We designed and fabricated a 3D printed PCL scaffold as an engineered periosteum that consists of a porous membrane constituted by PCL meshes. Without any treatment the mimetic periosteum facilitates the vascularization and formation of an induced membrane (IM) in vivo. After functionalization with rhBMP-2, the mimetic periosteum demonstrates osteoinductive properties in vitro and promotes highly efficient bone regeneration in vivo, reducing the effective dosage of the morphogen drastically. In addition, when functionalized with rhBMP-2 and combined with mesenchymal progenitor cells, the engineered periosteum supports delivery of both treatments into the damaged tissue.

Structurally, the periosteum is a fibrous, highly vascularized tissue that forms a suitable perivascular niche for osteochondroprogenitor and skeletal stem cells. These progenitors not only form the osteoblasts needed to produce bone tissue but also the signals that define the reparative response^27,56^. Engineering of periosteum has been approached with different printing and functionalization strategies through a variety of biocompatible fibrillar scaffolds, including PCL^37^, PEG-PLA^57^, PLGA^58^, type I collagen^59,60^, polydimethylsilolxane^61^ or polyurethane^62^.

PCL is a polymeric plastic material with low melting temperature allowing different printing approaches. Its high biocompatibility assures a complete absorption into the grafted tissue over time, producing a fully regenerated bone tissue. Three-dimensional melt electrowriting has gained recognition in the last few years for multiple reasons. Printing derived from electric field produces extremely thin fibers. Moreover, control over printing can be conducted by slightly varying pressure, voltage, or printing distance. Manufactured scaffolds can thus reach up to 4–5 mm in thickness (3D) allowing for a wide variety of architectures that can be easily tuned. Apart from this, extremely high porosity can be easily achieved^63^, conferring on these scaffolds the ability to host cells with free culture media diffusion. The successful printing of highly porous, thin, tubular PCL membrane provided us with the tool to replicate or mimic native periosteum from which we can customize and deliver any therapy in order to treat different bone defects. SEM results corroborated that our implants present microporosity, which allows further functionalization and creates an environment capable of retaining cells due to the high availability of surface area.

For a long time, vascularization has been understood as a key feature for proper regeneration, with most experts considering it to have a central role in functional osseous tissue^64^. Our design for a mimetic periosteum allows Masquelet-like induced membrane functionalization through ectopic implantation. Both ectopic and orthotopic implantation unchained a clear FBR against PCL meshes, as previously reported^37^. This was corroborated in our implants by the presence of CD68 positive, macrophage-derived giant multinucleated cells. We also discovered the presence of abundant neovascularization, with caveolin positive staining and mature vessel-like structures, through an αSMA positive staining. All these findings combined highlight the achievement of an induction membrane (IM) surrounding our PCL construct^65,66^.

The fact that different biological and cellular treatments (rhBMP-2, rPMSCs or mix of both) generate similar levels of vascularization clearly demonstrates that FBR against PCL is the main reason for IM formation. Despite results implying that the use of Masquelet´s would be successful, no healing was observed in any of the in vivo groups. Results from the orthotopic implantation after 10 weeks suggested that the first step for IM formation (6-week ectopic implantation) hinders a further therapeutic effect of either rPMSCs or rhBMP-2. The original Masquelet’s technique is a two-stage surgical approach for the treatment of critical-size defects, whereby a functional biological membrane is formed in response to a methyl methacrylate spacer. In a second stage, the spacer is removed and, after maintaining the integrity of the biological membrane, substituted by an appropriate osteoinductive material, originally cancellous bone autograft or a mix of cancellous bone autograft and allograft^67^. It is believed that the efficiency of the technique depends on the biological membrane which, once established, could deliver osteoinductive and vascular growth factors, suggesting that the biological membrane could function as a mimetic periosteum^68^. However, the contribution of the autograft cannot be ruled out as the principal driver of the regenerative process. Indeed, different approaches in rat models have been explored to define the therapeutic potential of the IM technique, demonstrating that a source of osteoinductive signal, in the form of autologous bone graft, is needed for successful bone regeneration^69–71^.

*BMP* signaling is considered to be one of the key pathways to initiate bone repair^56,72,73^. Though biologically BMP-2 is the molecule that triggers the reparative response, other BMPs (BMP-7, BMP-4, BMP-5, BMP-6, BMP-9) have been explored therapeutically or experimentally as alternative treatments in tissue engineering strategies for bone regeneration^74,75^.

Mimetic periosteum based on PCL MEW printing facilitates functionalization with PEA by radical polymerization^34^, showing the ability to absorb increasing amounts of rhBMP-2 when incubated in crescent concentrated solutions up to 100 µg/ml of rhBMP-2. Motivated by the fact that different authors have reported side effects related to usage of this morphogen at high supraphysiological doses^14,76,77^, we investigated rhBMP-2 functionalization studies using the two lowest doses that result in a bulk amount of rhBMP-2 that we could quantify by ELISA (10 µg/ml ≈ 55 ng; 25 µg/ml ≈ 190 ng). The kinetics of rhBMP-2 release showed that after 2 weeks 50% of the morphogen was still bound to the mimetic periosteum and that a sustained release profile supports the osteoblastic differentiation of human mesenchymal progenitor cells in vitro. Remarkably, the positive control dose was 190 ng of rhBMP-2 renewed on every media change (2–3 days), indicating that PEA coating enhances the presentation and effect of the long-lasting growth factor.

In vivo assays were carried out using critical-size femoral defects in rats, with an aluminum contact plate to provide tight fixation, prevent endochondral ossification, and ensure that newly formed bone was derived from intramembranous ossification. The in vivo model presented in this paper has shown that our regenerative approach represents an improvement in the application of rhBMP-2 in comparison with previous studies using this morphogen. Models using ACS as a delivery system use amounts of rhBMP-2 between 11 and 5 μg of rhBMP-2 for orthotopic implantation, with good results in terms of healing and mechanical competence of the repaired bone^78–80^. Other platforms for rhBMP-2 delivery, such as collagen microspheres^81^ or nanofiber mesh alginate hydrogel^82^, have reduced the effective rhBMP-2 dosing to 1–3 μg when evaluated orthotopically on a femoral (nanofiber mesh alginate, 1 μg) or a calvaria defect (microspheres, 3 μg). We have demonstrated that 55 ng of rhBMP-2 is sufficient to produce effective repair of a critical-size defect of the tibia and optimal regeneration is obtained when the rhBMP-2 is increased to 190 ng of rhBMP-2.

In conclusion, here we demonstrate that 3D printed engineered periosteum is susceptible to being functionalized with different approaches, demonstrating function as a native periosteum by delivering a key osteogenic growth factor and significant progenitor cells into a large bone defect. The easy manufacture system, potential for upscale, capacity for long-term storage, and possibility of personalization all suggest that our mimetic periosteum design has a high therapeutic and translational potential.

## Methods

### Fabrication and coating of implants

Medical graded 50 kDa poly ε-caprolactone (PCL, PURASORBPC 12, Corbion Purac, The Netherlands) was used to synthesize the whole implant. Cylindrical core PCL scaffolds were obtained using a 4 mm punch from PCL sheets generated using additive manufacturing with a 0–90° lay-down pattern and 2 mm pore size.

Outer membranous scaffolds of PCL were free solvent printed using melt electrospinning writing technology (MEW; Queensland University of Technology, Australia) with the following parameters: 2.5 bars, 6 kV, 2750 mm/min linear speed, 450 rad/min angular speed and 6 mm printing distance from collector. In order to reduce the membrane hydrophobicity, outer membranous PCL scaffolds were treated with O_2_/Ar plasma for 8 min (Diener electronic, Plasma-surface-technology, Ebhausen, Germany). Afterwards, PCL membranes were coated with poly-ethyl acrylate (PEA). PEA was obtained by radical polymerization using benzoin (98% pure; Scharlau) as a photoinitiator as described previously^34^.

### Characterization of rhBMP-2 dosing and release profile

To allow the adsorption of rhBMP-2 to the PCL-PEA scaffolds, human fibronectin (hFN, R&D systems) was used as an intermediate binding molecule. For coating the membranes with rhBMP-2, after PEA treatment, membranes were incubated in a solution of hFN (20 µg/ml) for 1 h at room temperature. The excess of non-adhered hFN was washed out using PBS, the materials were then dried with a sterile gauze. Finally, scaffolds were incubated in different concentrations of rhBMP-2 (InductOs 12 mg, Medtronic BioPharma B. V., Minneapolis, MN, USA) for 1 h at room temperature. The excess of rhBMP-2 was removed with a washing step in PBS. To assess net dosage load, rhBMP-2 was released from the membranes by incubating with a PBS solution containing 0.05% w/v of SDS for 30 min at 90 °C in a Thermoblock.

In addition, the release profile of specific rhBMP-2 loaded PCL-PEA-FN membranes was also determined. Membranes were loaded with each specific concentration and placed in separated Eppendorf tubes with 500 µl of PBS. To simulate physiological conditions tubes were incubated at 37 °C in continuous orbital agitation at 10 rpm using an incubator (Labnet Biotecnica SL, Spain) for a total period of 15 days. 100 µl of each supernatant were collected at days 1, 5, 8, 11 and 15. To evaluate if the total amount of the obtained rhBMP-2 matched the expected net dose of rhBMP-2, the remaining rhBMP-2 attached into the scaffolds after 15 days was released as described previously.

The quantification of rhBMP-2 from all supernatants was measured by ELISA following the manufacturer instructions (RayBio® Human BMP-2 ELISA kit, Raybiotech, Norcross, GA, USA).

### Isolation of periosteal mesenchymal stem cells

Rat periosteal mesenchymal stem cells (rPMSCs) were extracted from hind limbs of transgenic Sprague-Dawley rats containing the fluorescent protein GFP under the control of ubiquitin-C promoter (SD-TgGFP)^83,84^. After euthanizing the animals employing carbon dioxide (CO_2_), femurs and tibias were isolated and cleaned of muscle tissue gently, to avoid damaging the periosteum. Periosteal explants were obtained by scratching the surface of the hindlimbs long-bones with a surgical blade and directly seeding the explants into six-well plates (Corning, Corning, NY, USA) for expansion. rPMSCs were cultured in expansion medium, Dulbecco´s Modified Eagle Medium (DMEM) supplemented with 10% fetal bovine serum (Gibco, Thermo Fisher Scientific, Waltham, MA, USA), 1% Penicillin/Streptomycin (P/S) (Gibco, Thermo Fisher Scientific) and basic fibroblast growth factor (bFGF) 10 ng/ml (Peprotech, Thermo Fisher Scientific). Cells were expanded and used in our experiments between passages 1–3.

Human periosteal mesenchymal stem cells (hPMSCs) were also isolated from human periostea obtained during anterior cruciate ligament (ACL) reconstruction procedures after written informed consent in the Clínica Universidad de Navarra (CEI 029/13)^27^. Periosteal explants were carefully washed with PBS, minced, and plated into six-well plates using expansion medium. All experiments involving hPMSCs were performed with passage 2–3 cells.

Phenotypic mesenchymal characterization for rPMSCs and hPMSCs has been previously described^27,42^.

### In vitro osteogenic differentiation

Human derived PMSCs from five different patients were differentiated into osteoblastic lineage within the PCL-PEA porous scaffolds. The PCL-PEA membranes were treated by washing with ethanol 70% w/v and by exposure to ultraviolet light for 30 min. Following this, scaffolds were functionalized with hFN and rhBMP-2 (25 µg/ml) under a laminar flow hood. Osteogenic differentiation was carried out in 24 well plates. A total of 250,000 hPMSCs were seeded in each well or scaffold. Plates were then placed into a cell culture incubator for 3 h, allowing hPMSCs to adhere to the plates or the membranes. Finally, every well was topped up with 500 µl of DMEM supplemented with 10% FBS and 1% P/S. Following 24 h of incubation after seeding, the process of differentiation into osteoblastic lineage began. Negative control groups were maintained in DMEM supplemented with 10% FBS and 1% P/S along the differentiation period, whereas in experimental differentiation wells the medium was changed to differentiation medium (DMEM high glucose supplemented with 10% FBS, 1% P/S, 50 µg/ml ascorbic acid (Sigma-Aldrich, St. Louis, MO, USA), β-glycerophosphate 10 mM (Sigma-Aldrich) and 10 nM dexamethasone (Sigma-Aldrich). In the positive control groups, the differentiation medium was supplemented with 200 ng of rhBMP-2, and all culture medium was changed every 2-3 days. Cells underwent differentiation for 21 days and samples were collected at days 0, 7, 14 and 21.

To assess osteoblastic differentiation, after 21 days, cultures were subjected to Alizarin Red staining. Each well was carefully washed with PBS and then fixed with formalin 10% (Panreac) during 10 min RT. Formalin was then removed and washed with PBS and wells were incubated with 2% Alizarin Red solution, pH 4.1, and incubated for 15 min at RT. Finally, wells were gently washed up with distilled water twice and pictures were taken.

### Gene expression analysis

Cell samples were collected on days 7, 14 and 21 and subjected to RNA extraction using a standard Trizol (Sigma Aldrich) protocol. Isolated RNAs were quantified using a Nanodrop^TM^ and all samples included in the analysis had a minimum RNA concentration of 50 ng/µl. Between 200 ng and 1 μg of total RNA was reverse transcribed to cDNA using cDNA Qscript mix (Quantabio, Massachusetts) in compliance with manufacturer instructions. Quantification was carried out by qPCR with Taqman probes using *RPLP0* (Hs99999902_m1) as control. We assessed the differential expression of *COL1A1* (Hs00164004_m1), *RUNX2* (Hs00231692_m1), *BGLAP* (Hs01587814_g1) and *SPP1* (Hs00960942_m1). Amplification curves and CT values were analyzed using QuantStudio Design and Analysis software (Thermo Fisher Scientific), relative gene expression levels were determined using the 2^−ΔΔCT^ method.

### Femoral critical-size bone defect

All experiments involving animals were approved by the Ethics Committee for Animal Experimentation of the University of Navarra (Comité de Ética para la Experimentación Animal, CEEA) and Navarra regional Government, CEEA# 105-17 and CEEA# 073-20. Eight- to twelve-week-old female Sprague Dawley rats (Harlan Laboratories Inc, Indianapolis, IN) underwent surgery to create a critical-size diaphyseal defect (5 mm) in the right femur as previously described^42^. The animals were anesthetized with isoflurane and shaved. A wound of 20 mm was opened, the muscle was separated, and the femur was carefully exposed. A custom aluminum plate (20 mm long × 4 mm wide × 2 mm high) was fixed to the femur using 4 screws (8 mm long × 1.5 mm diameter) to provide mechanical stabilization. A 5 mm defect was generated with a surgical bur in the diaphysis and by placing the specific treatment in the bone gap. Following the ethical criteria, it was performed an analgesic protocol with an intraperitoneal injection of Fentanyl (300 μg /Kg), a subcutaneous injection of Ketoprofen (5 mg/Kg) for inflammation during surgery followed by a subcutaneous injection of Buprenorphine (50 μg /Kg) every 12 h for 48 h for pain relief.

When rPMSCs were applied, 3 million cells were seeded 24 h pre-surgery, or in the operating room right before scaffold implantation. Rats undergoing surgery were then allowed to heal for 10 weeks and euthanized with carbon dioxide (CO_2_). Every in vivo group described was composed of at least *n* = 6 animals. Groups receiving rPMSCs were also analyzed at weeks 1, 3 and 5 post-surgery to assess cellular integration.

### Ectopic implantation

To produce induced membrane-like implants, PCL implants were ectopically implanted subcutaneously in the back of Sprague-Dawley rats. Animals receiving implants (4–6 implants per animal) were anesthetized with isoflurane, the back area was shaved, cleansed with povidone and H_2_O_2_ and a small incision was opened with a surgical blade. Metzenbaum scissors were utilized to detach the skin and implants were grafted. The pain after was controlled with a subcutaneous injection of Buprenorphine (50 μg /Kg) every 12 h for 48 h. Constructs were implanted subcutaneously for a total of six weeks to generate a foreign body reaction over the PCL scaffolds. After 6 weeks, the animals were sacrificed, and implants were recovered and were either used as individual experimental implants in the critical-size defect model previously described or processed for histological analysis.

### Radiographic and micro-computed tomography analysis

The bone healing process was followed up by radiographic analysis. Single-plane X-ray images were taken at weeks 1, 3, 5 and 7 after surgery using an X-ray micro-CT (Quantum-GX, Perkin Elmer, Waltham, MA, USA). Radiographic assessment was performed using a simplified radiographic union score tibia (RUST) at 7-weeks post-surgery, classifying the defects into three different groups; Score = 2, the highest score, meaning continuous cortical healing; Score = 1, the repaired defect showing bone growth in the defect, but a fracture line was present; Score = 0, where the defect showed no significant bone production, and a gap was visible (Supplementary Fig. 9).

µCT analyses were performed 10 weeks after surgery. Full femoral 3D tomographic images were acquired using X-ray micro-CT (Quantum-GX) at 90 kVp X-ray source voltage, 88 μA current, and the high-resolution scan protocol for a total acquisition time of 14 min and a gantry rotation of 360 degrees. The tomographic images containing the whole bone had a total of 512 slices with isotropic 50 μm voxel size and a resolution of 512 × 512 pixels per slice. Three-dimensional reconstruction was carried out using the AMIRA software (FEI-Thermo Fisher Scientific, USA). The noise was filtered, and the data was calibrated and segmented at a threshold of 1200 Hounsfield units (soft mineralized tissue <1200; mineralized tissue >1200).

### Histology, immunohistochemistry, and immunofluorescence

Right hind limbs were extracted from the hip joint and fixed with 10% buffered formalin (Panreac-ITW Reagents, Darmstadt, Germany) for 48 h. After µCT analyses, femurs were carefully cleaned of the muscle and submerged in an EDTA-PVP decalcifying solution (10% EDTA, 7.5% polyvinylpyrrolidone, 0.1 M Tris, pH 6.95) for 8 weeks. Samples were further dehydrated in graded ethanol and xylene and embedded in paraffin. Four µm sections were serially cut in the coronal plane in each sample, selecting the femur core sections for the histological analysis. Hematoxylin and Eosin (H&E) staining was then used for general evaluation.

For immunohistochemistry, sections were subjected to antigen retrieval using Tris-EDTA pH 9.0 solution and a pressure cooker. Slides were then incubated with primary antibodies, anti-green fluorescent protein (GFP) (1:2000), (NB100-1770S, Novus Biologicals), anti CD68 (1:50) (ab31630, Abcam, Cambridge, UK), anti PRRX1 (1:50) (HPA063566, Sigma) and anti-type I collagen (COL1) (1:100) (H3884, Sigma). Cool overnight at 4 °C in a humidity chamber. Staining was developed by peroxidase with diaminobenzidine (DAB) in line with manufacturer instructions (EnVision, Dako, Glostrup, Denmark).

For Immunofluorescence staining, antigen retrieval was performed using trypsin (4 mg/ml in PBS supplemented with 1 mM CaCl_2_) for 1 hr at 37 °C within a humidity chamber. Non-specific sites were blocked with 5% BSA solution (A-4503, Sigma-Aldrich) in a humidity chamber at RT and finally sections were incubated with primary antibodies overnight at 4 °C using a humidity chamber. Primary antibodies used were anti smooth muscle actin (αSMA) (A-2547, Sigma Aldrich) and anti-caveolin (3238S, Cell Signaling Technology) in a dilution of 1:1000 and 1:100 respectively. After incubation, primary antibodies were washed with PBS and samples were incubated with secondary fluorescent antibodies (Alexa Fluor®488, A11029; Alexa Fluor®568, A11036; Invitrogen, Thermo Fisher). Finally, secondary antibodies were washed with PBS for 5 min. For nuclear staining, DAPI (H-1200, Vectashield®, Vector Laboratories, Burlingame, CA, USA) was added with the mounting medium (S3023, Dako).

Immunofluorescence signals were quantified using ImageJ/Fiji software. (*n* = 6 animals per group). The average signal was measured as the total area and expressed in arbitrary units.

Illustrations summarizing each project part were created using BioRender© (https://app.biorender.com). Illustrations and digital images were imported into Adobe Photoshop and formatted into journal standards.

### Biomechanical testing

Biomechanical testing was performed to assess the quality and mechanical properties of newly regenerated bone using an Instron 8874 (Instron, Norwood, MA). Both femoral tips were embedded in resin, Demotec 30, (Demotec, Nidderau, Germany) and placed in custom molds. One femur end (metaphysis) remained fixed during the mechanical test, whereas the other end was forced to rotate in an internal direction at a speed of 1/360 Hz until fracture. The ultimate failure of the tested bones was achieved by only applying torsion strength, as the load placement was set to 0 and carefully monitored so that no tension or compression strength could be applied. In order to mimic the physiological environment, bones were kept moisturized by surrounding them with a soaked gauze. Maximum torque and failure angle data from this process were recorded at 100 Hz. Additionally, the maximum torque displayed by the treated femur relative to its control allowed computation of the repair rate.

### Statistical analysis

All the results obtained were expressed as a median with an interquartile range, whiskers representing minimum and maximum values. GraphPad Prism 9.4.0 software (GraphPad Software Inc, La Jolla, CA) was used for statistical analyses. The normality and test distribution parameters of the different data sets were evaluated by a Kolmogorov-Smirnov test. Statistical significance was determined by the ANOVA or Kruskal–Wallis test followed by a post hoc analysis test according to the normal distribution of the data analyzed. Significance was set at *p* < 0.05.

### Reporting summary

Further information on research design is available in the Nature Research Reporting Summary linked to this article.

### Supplementary information

Supplemental Information

Reporting Summary

## Data Availability

The authors will make the data supporting the findings of this study available on reasonable request to the corresponding authors.
