# Supplementary material for: An engineered periosteum for efficient delivery of rhBMP-2 and mesenchymal progenitor cells during bone regeneration
Source: NPJ Regen Med. 2023 Sep 29;8:54. doi: 10.1038/s41536-023-00330-2 (PMC10541910; doi:10.1038/s41536-023-00330-2)
Supplement: Supplementary file 1 — Supplemental Information [file 41536_2023_330_MOESM1_ESM.pdf]

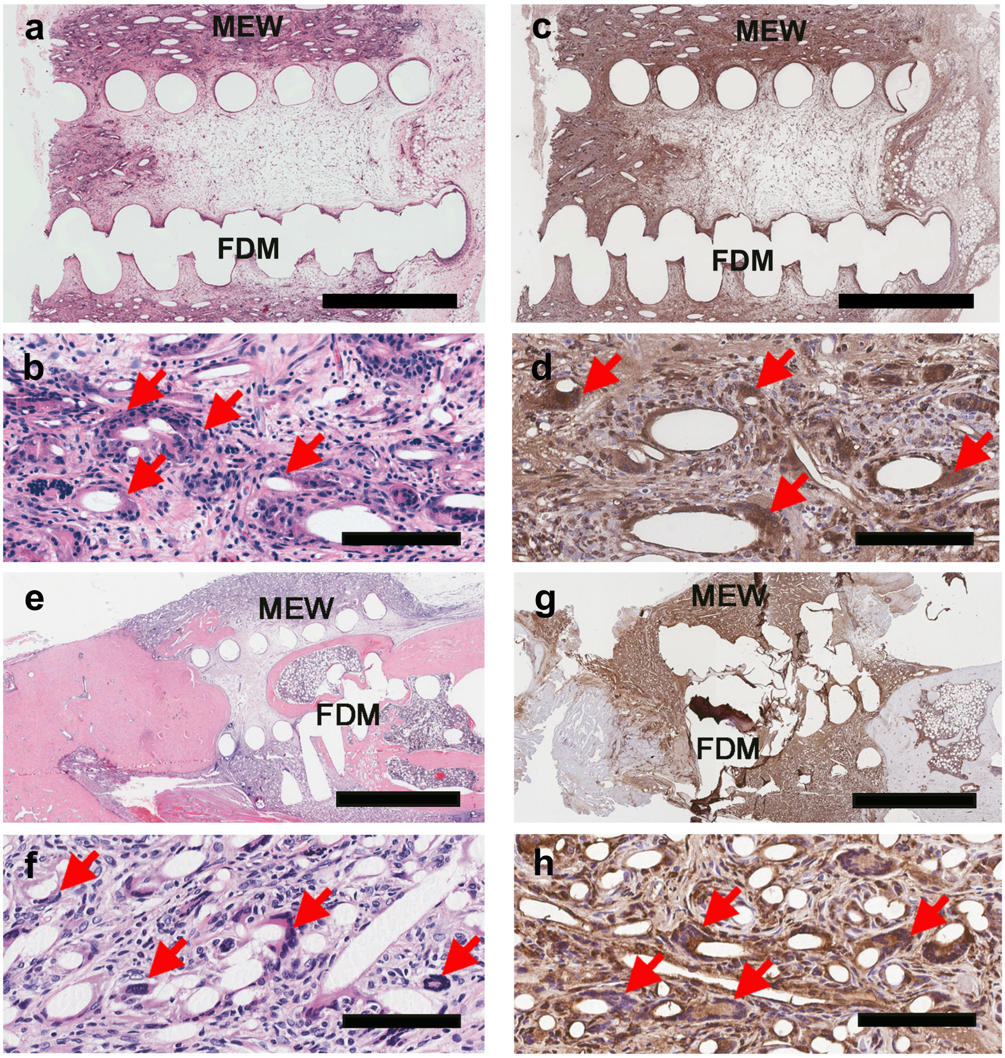

Supplemental figure 1. Histological and immunohistological evaluation of the foreign body reaction generated by the mimetic periosteum. **a,** Whole histological view, H&E staining, of the implant after 6 weeks of ectopic subcutaneous implantation. FDM, inner PCL scaffold; MEW, mimetic periosteum, outer PCL scaffold. Scale bar = 1.5 mm. **b,** Magnification of the histological preparation centered on the mimetic periosteum (MEW scaffold). Arrow heads pointing at multinucleated giant cells. Scale bar = 100 µm. **c,** Whole view of the reactivity of the implant to anti CD68 antibody; scale bar = 2 mm. **d,** magnification of the implant showing that anti CD68 reactivity centered in the mimetic periosteum (MEW scaffold). Scale bar = 100 µm. **e,** Whole histological view, H&E staining, of the defect area after 10 weeks of orthotopic implantation. Scale bar = 1.5 mm. **f,** Magnification of the implant centered on the mimetic periosteum (MEW). Arrow heads pointing at multinucleated giant cells. Scale bar = 100 µm. **g,** Whole view of the reactivity of the implant to anti CD68 antibody. Scale bar = 2 mm. h, Magnification of the mimetic periosteum showing anti CD68 reactivity to multinucleated giant cells. Scale bar = 100 µm.

XRs images	Scoring
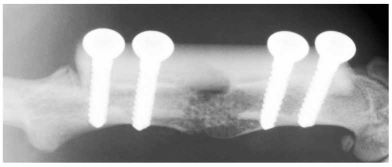	**2**
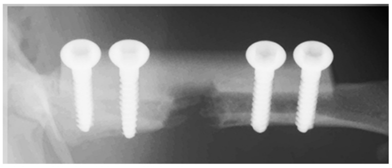	**1**
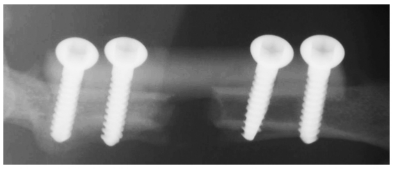	**0**

Supplemental figure 2. RUST scoring assessed by radiographic imaging of 7 weeks post-surgery femurs. Score = 2, full healing; Score = 1, substantial bone growth but no healing, a fracture line is visible; Score = 0, no substantial bone growth, and a gap is visible.

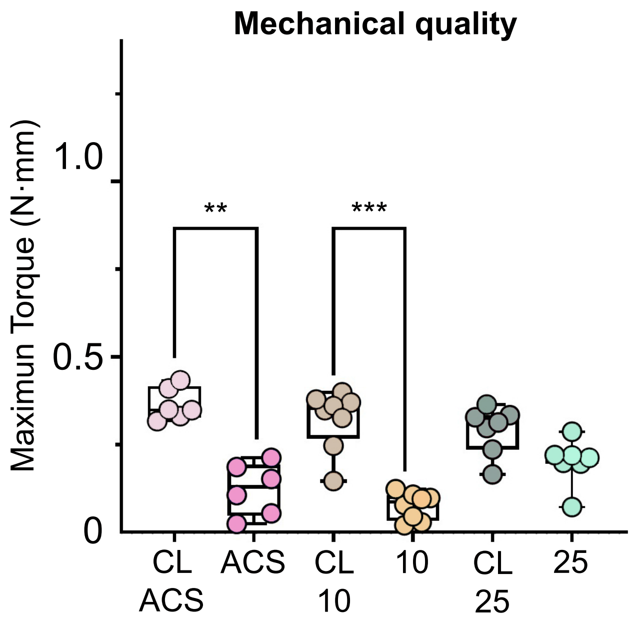

Supplemental figure 3. Mechanical assessment of the regenerated bone and contralateral (CL) femurs. Maximum torque (N mm) of the treated (ACS, 190 ng of rhBMP-2 delivered with absorbable collagen sponge, n = 6; 10, 55 ng of rhBMP-2 delivered with PCL MEW, n = 8; 25, 190 ng of rhBMP-2 delivered with PCL MEW, n = 7) and intact, CL bones. Statistical significance was determined by Kruskal-Wallis test ($p < 0.001$) followed by Dunn's multiple comparisons test. **, $p = 0.0021$; ***, $p = 0.0002$. Results are expressed as a median with an interquartile range, whiskers representing minimum and maximum values.

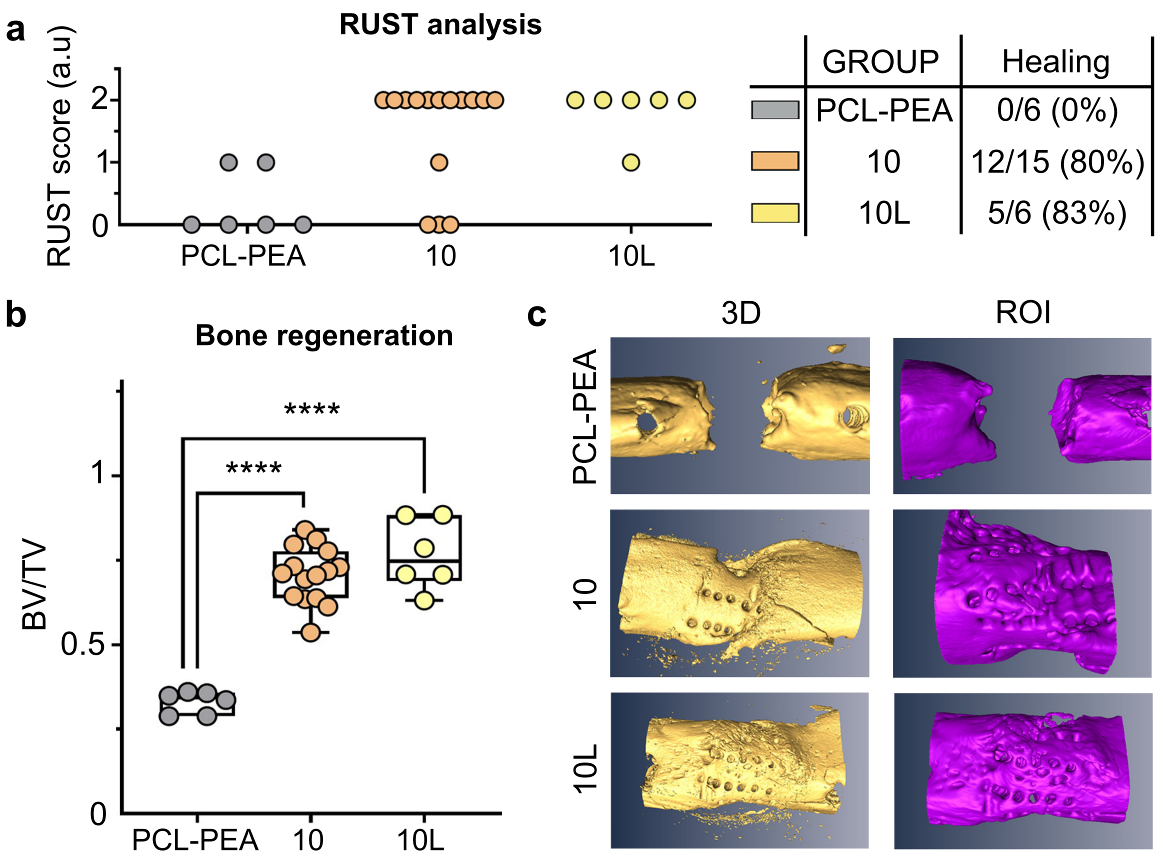

Supplemental figure 4. Efficiency of bone regeneration for lyophilized mimetic periosteum. **a,** Radiographic scoring, and healing assessment. PCL-PEA, non-functionalised PCL implant (n = 6); 10, PCL implant with outer MEW functionalised with 10 µg/ml of rhBMP-2 (n = 15); 10L, PCL implant with outer MEW functionalised with10 µg/ml of rhBMP-2 and lyophilized before assembly (n = 6). **b,** Quantification of newly regenerated bone (BV/TV) at the ROI. Statistical significance was determined by one way ANOVA (F = 57.59, $p < 0.0001$) followed by Tukey's multiple comparison test. ****, $p < 0.0001$. **c,** Three-dimensional rendering of the regenerated bone at the ROI. Results expressed as a median with an interquartile range, whiskers representing minimum and maximum values. expressed as a median with an interquartile range, whiskers representing minimum and maximum values.

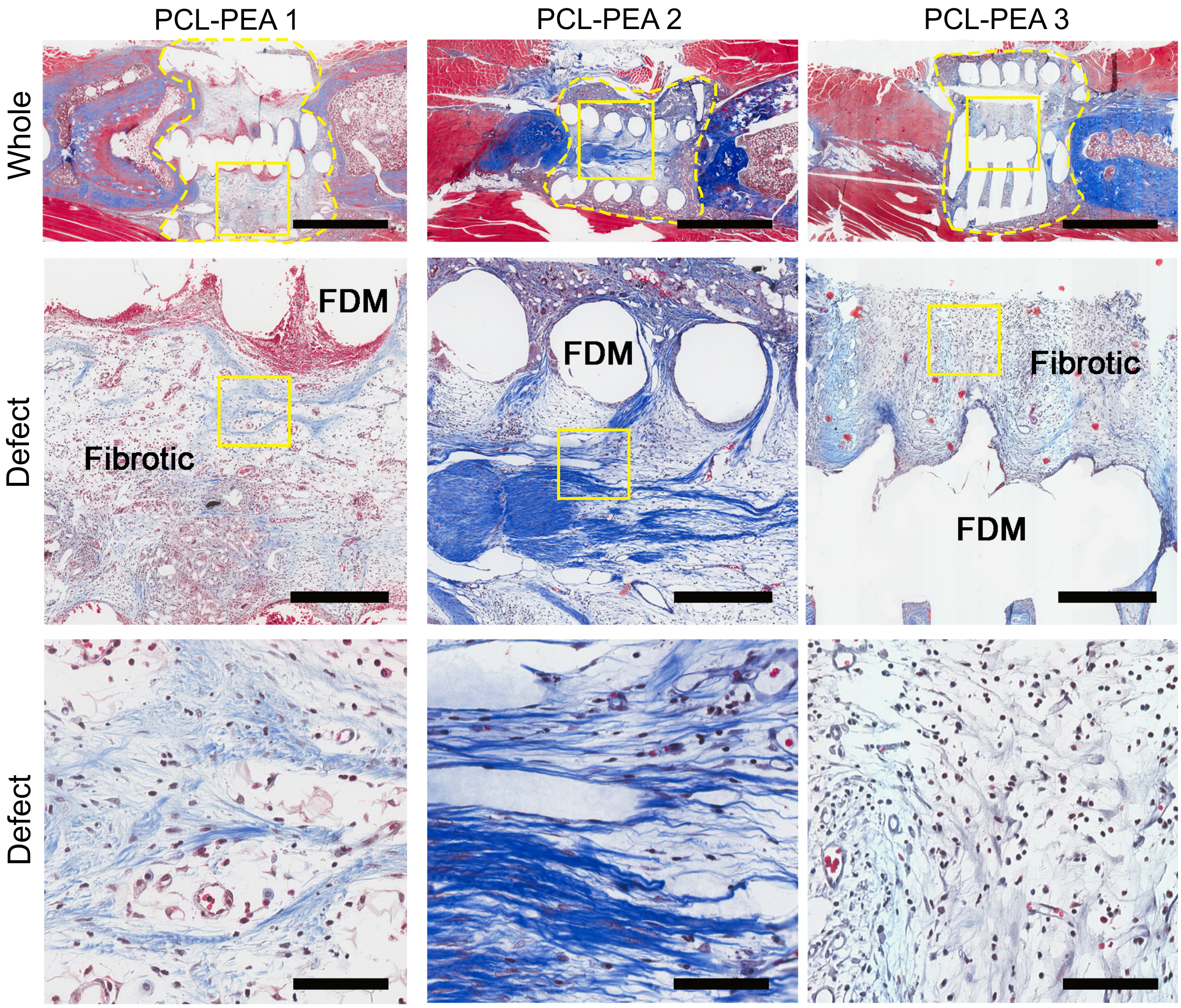

Supplemental figure 5. Masson's trichrome staining of different samples from control group. Upper row (Whole), general view of the ROI with yellow squares defining magnification areas, scale bar = 2 mm. Middle row (Defect), magnification of the defect area MT stained showing fibrotic tissue, scale bar = 400 µm. Lower row (Defect), increased magnification of the defect area, scale bar = 50 µm.

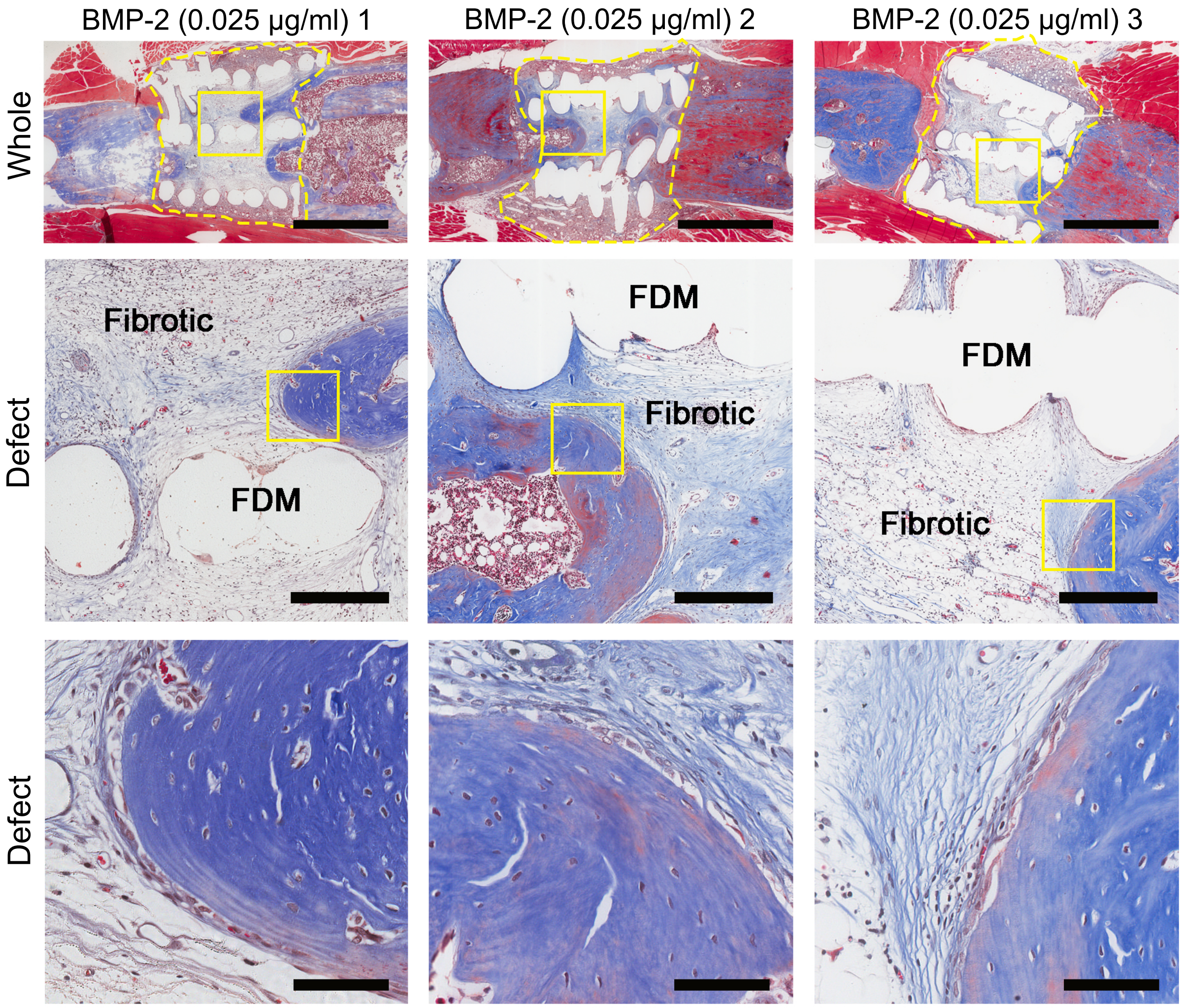

Supplemental figure 6. Masson's trichrome staining of different samples from 0.025 µg/ml rhBMP-2 group. Upper row (Whole), general view of the ROI with yellow squares defining magnification areas, scale bar = 2 mm. Middle row (Defect), magnification of the defect area MT stained showing fibrotic tissue and newly regenerated bone MT stained in blue, scale bar = 400 µm. Lower row (Defect), increased magnification of the defect area, scale bar = 50 µm.

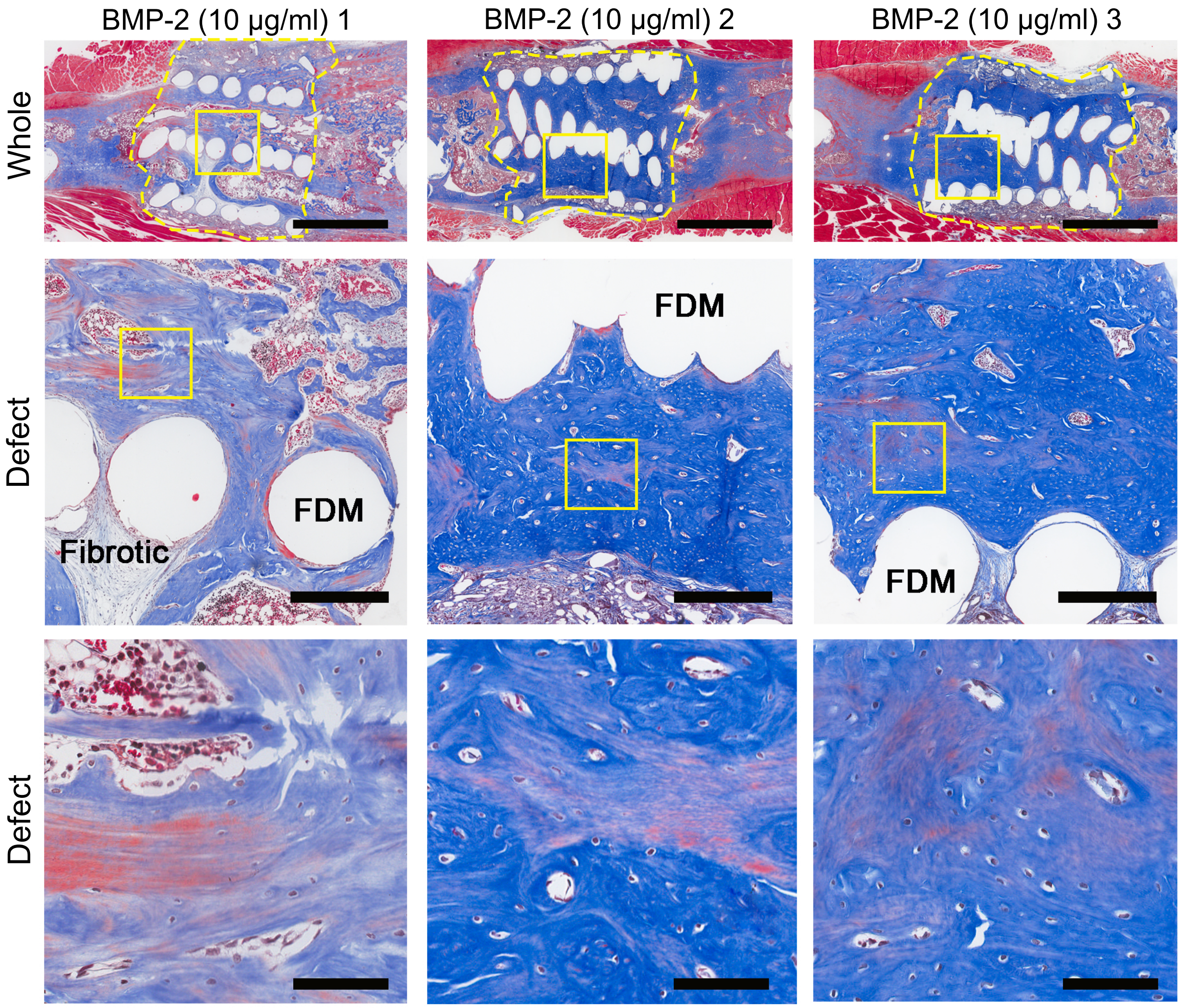

Supplemental figure 7. Masson's trichrome staining of different samples from 10 µg/ml rhBMP-2 group. Upper row (Whole), general view of the ROI with yellow squares defining magnification areas, scale bar = 2 mm. Middle row (Defect), magnification of the defect area MT stained showing newly regenerated bone MT stained in blue and some reddish areas, scale bar = 400 µm. Lower row (Defect), increased magnification of the newly formed bone within the defect area, scale bar = 50 µm.

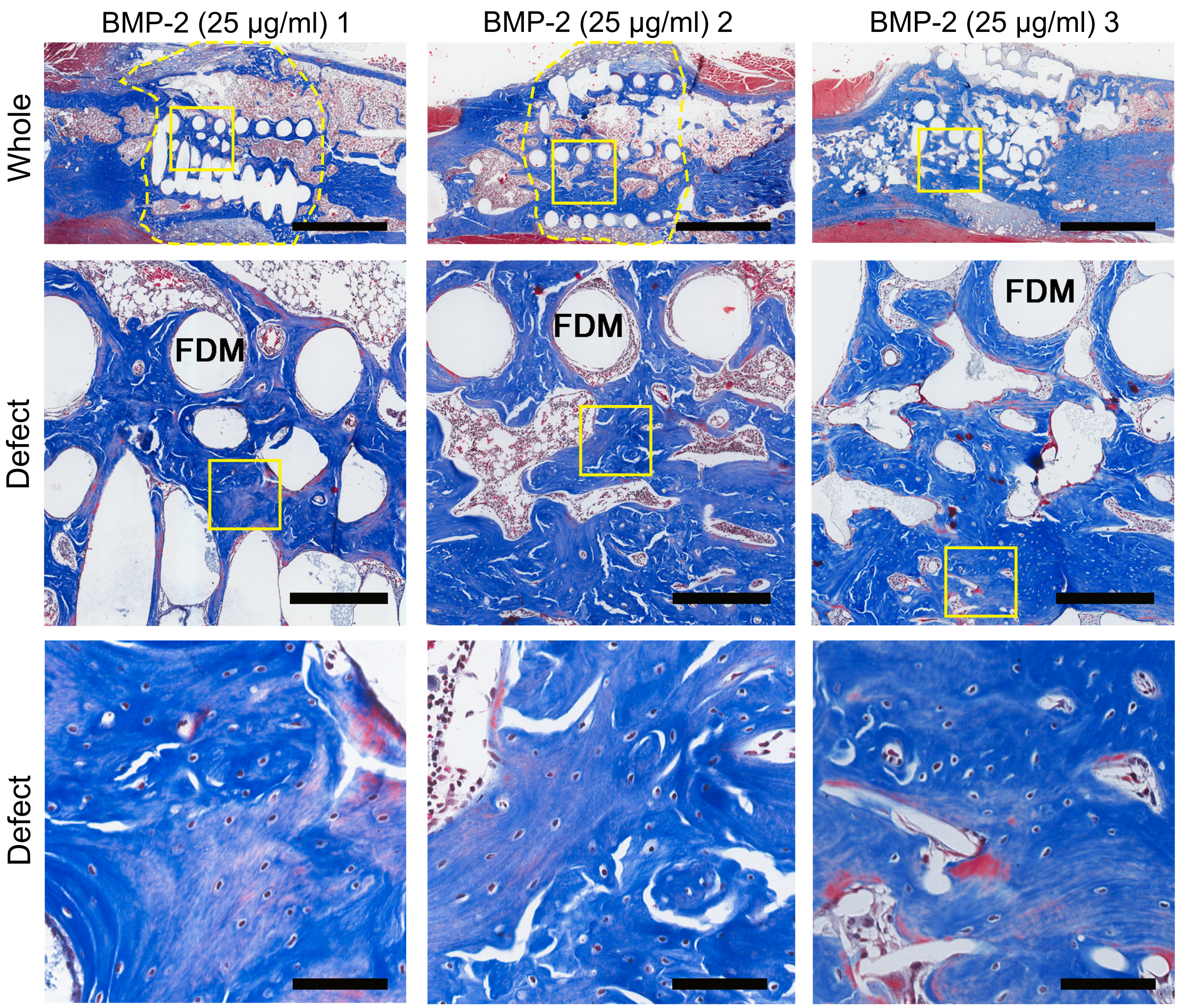

Supplemental figure 8. Masson's trichrome staining of different samples from 25 µg/ml rhBMP-2 group. Upper row (Whole), general view of the ROI with yellow squares defining magnification areas, scale bar = 2 mm. Middle row (Defect), magnification of the defect area MT stained showing newly regenerated bone MT stained in blue and some reddish areas, scale bar = 400 µm. Lower row (Defect), increased magnification of the newly formed bone within the defect area, scale bar = 50 µm.

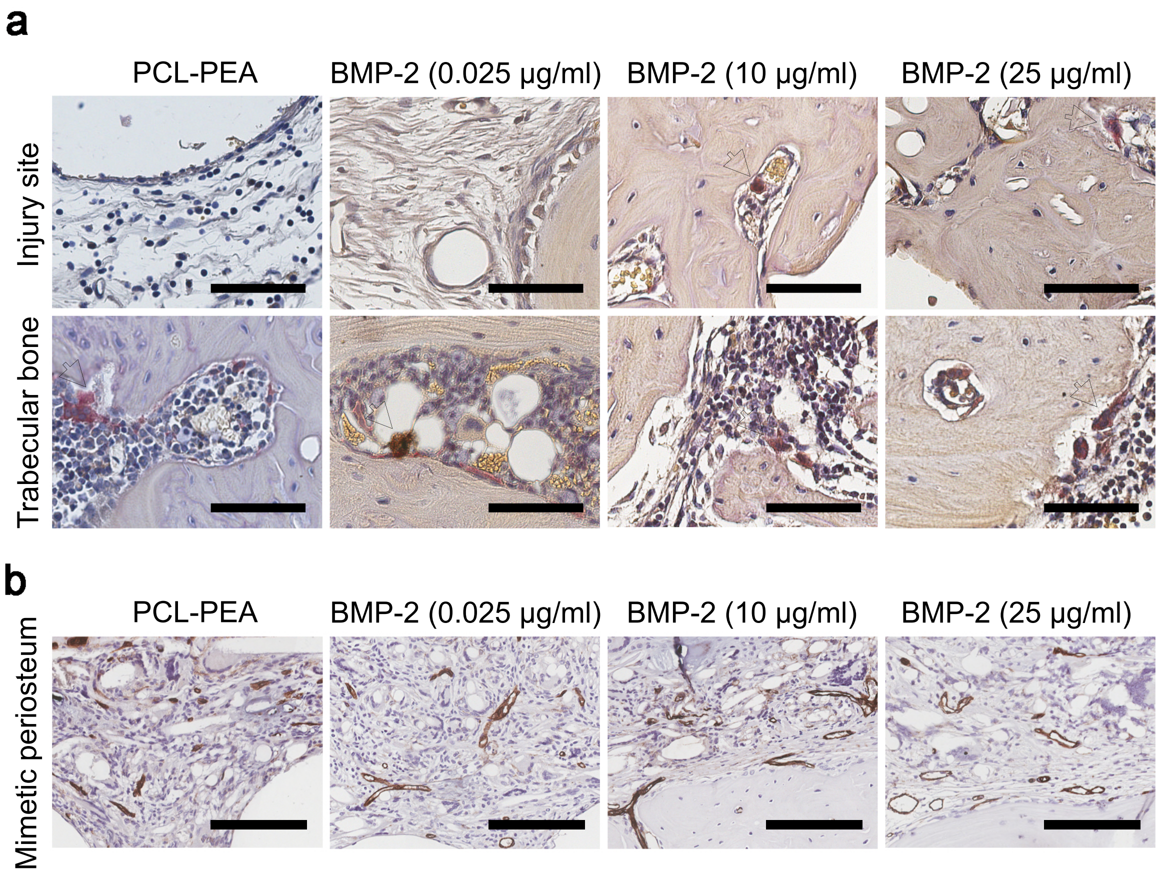

Supplemental figure 9. Histo and immunohistochemistry for further characterizaton of mature bone biology. **a,** TRAP staining for both, injury site (upper rows) and trabecular bone (lower rows) of the non-functionalised implants (PCL-PEA) or functionalized with different concentrations of rhBMP-2. Red arrow heads pointing at labeled osteoclasts. Scale bars = 75 µm. **b,** Immunostaining of Cav (caveolin) showing vasculature formation within newly formed mimetic periosteum. Scale bars = 150 µm.
