# Supplementary material for: An engineered periosteum for efficient delivery of rhBMP-2 and mesenchymal progenitor cells during bone regeneration
Source: NPJ Regen Med. 2023 Sep 29;8:54. doi: 10.1038/s41536-023-00330-2 (PMC10541910; doi:10.1038/s41536-023-00330-2)
Supplement: Supplementary file 2 — Reporting Summary [file 41536_2023_330_MOESM2_ESM.pdf]

nature portfolio

Corresponding author(s): Emma Muiños-Lopez & Froilán Granero-Moltó

Last updated by author(s): Jun 7, 2023

Reporting Summary

Nature Portfolio wishes to improve the reproducibility of the work that we publish. This form provides structure for consistency and transparency in reporting. For further information on Nature Portfolio policies, see our Editorial Policies and the Editorial Policy Checklist.

Statistics

For all statistical analyses, confirm that the following items are present in the figure legend, table legend, main text, or Methods section.

n/a	Confirmed	
☐	☒	The exact sample size (*n*) for each experimental group/condition, given as a discrete number and unit of measurement
☒	☐	A statement on whether measurements were taken from distinct samples or whether the same sample was measured repeatedly
☐	☒	The statistical test(s) used AND whether they are one- or two-sided<br>*Only common tests should be described solely by name; describe more complex techniques in the Methods section.*
☒	☐	A description of all covariates tested
☐	☒	A description of any assumptions or corrections, such as tests of normality and adjustment for multiple comparisons
☐	☒	A full description of the statistical parameters including central tendency (e.g. means) or other basic estimates (e.g. regression coefficient) AND variation (e.g. standard deviation) or associated estimates of uncertainty (e.g. confidence intervals)
☐	☒	For null hypothesis testing, the test statistic (e.g. *F*, *t*, *r*) with confidence intervals, effect sizes, degrees of freedom and *P* value noted<br>*Give P values as exact values whenever suitable.*
☒	☐	For Bayesian analysis, information on the choice of priors and Markov chain Monte Carlo settings
☒	☐	For hierarchical and complex designs, identification of the appropriate level for tests and full reporting of outcomes
☒	☐	Estimates of effect sizes (e.g. Cohen's *d*, Pearson's *r*), indicating how they were calculated

Our web collection on statistics for biologists contains articles on many of the points above.

Software and code

Policy information about availability of computer code

Data collection	Scanning electron microscopy images were collected using ZEISS Gemini FE-SEM (Field Emission Scanning Electron Microscopy)<br>Histology images were collected with the Aperio Scaner CS2 (Leica) and immunofluorescence Images with the Vectra Polaris (Perkin Elmer)<br>Differentiation analysis images were performed with the magnifier Olympus SZ61.<br>Single-plane X-ray images and computed axial tomography were taken using Quantum GX microCT (Perkin Elmer)<br>Immunofluorescence images were quantified using ImageJ/Fiji software<br>Mechanical properties of newly regenerated bone were collected using an Instron 8874 (Instron, Norwood, MA)<br>CT values of the gene expression for the differentiation were obtained using QuantStudio Design (Thermo Fisher Scientific)
Data analysis	The software for the analysis of the scaned electron microscopy images was SmartSEM (ZEISS)<br>For the treatment of the histological images, the INT-SS-0157-Rev (V12.4.3.500.8) (Leica) software was used<br>Amira V5.2 (Thermo Fisher) was used for the treatment and analysis of bone regeneration images<br>GraphPad Prism (V9.3.1) software was use for all statistical analysis<br>Analysis of gene expression for the differentiation were obtained using the QuantStudio Design Analysis software (Thermo Fisher Scientific)

For manuscripts utilizing custom algorithms or software that are central to the research but not yet described in published literature, software must be made available to editors and reviewers. We strongly encourage code deposition in a community repository (e.g. GitHub). See the Nature Portfolio guidelines for submitting code & software for further information.

Data

Policy information about availability of data

All manuscripts must include a data availability statement. This statement should provide the following information, where applicable:

- Accession codes, unique identifiers, or web links for publicly available datasets
- A description of any restrictions on data availability
- For clinical datasets or third party data, please ensure that the statement adheres to our policy

Raw data for human dataset are not publicly available to preserve individuals' privacy under the European General Data Protection Regulation. However, the authors declare that the data supporting the findings of this study are available after reasonable request to the corresponding authors.

Research involving human participants, their data, or biological material

Policy information about studies with human participants or human data. See also policy information about sex, gender (identity/presentation), and sexual orientation and race, ethnicity and racism.

Reporting on sex and gender	Patients from both sex were collected.
Reporting on race, ethnicity, or other socially relevant groupings	No relevant grouping related with race, ethnicity or others was used to select the participants to harvest the samples on this study.
Population characteristics	People suffer from anterior cruciate ligament (ACL) reconstruction procedures
Recruitment	The samples used for this studies were stored on the Biobank until the development of the experiments and they were obtained between 2018 and 2020.
Ethics oversight	Human periosteal mesenchymal stem cells (hPMSCs) were isolated after written informed consent and in accordance with the regulations of the Review Board of Clínica Universidad de Navarra under the protocol CEI 029/13.

Note that full information on the approval of the study protocol must also be provided in the manuscript.

Field-specific reporting

Please select the one below that is the best fit for your research. If you are not sure, read the appropriate sections before making your selection.

☒ Life sciences ☐ Behavioural & social sciences ☐ Ecological, evolutionary & environmental sciences

For a reference copy of the document with all sections, see nature.com/documents/nr-reporting-summary-flat.pdf

Life sciences study design

All studies must disclose on these points even when the disclosure is negative.

Sample size	Sample size was determined according with previous results from similar studies. In general, in vivo models included between 6 to 15 individuals.
Data exclusions	No data were excluded from the analysis
Replication	Technical duplicates were used in gene expression analysis and mimetic periosteum functionalization.
Randomization	Age-matched mice were randomly distributed into experimental groups. Only female rats were used in the experiments of this study.
Blinding	All the samples included in this work were pseudo-anonymized to perform non-vias analysis.

Reporting for specific materials, systems and methods

We require information from authors about some types of materials, experimental systems and methods used in many studies. Here, indicate whether each material, system or method listed is relevant to your study. If you are not sure if a list item applies to your research, read the appropriate section before selecting a response.

Materials & experimental systems

n/a	Involved in the study	
☐	☒	Antibodies
☒	☐	Eukaryotic cell lines
☒	☐	Palaeontology and archaeology
☐	☒	Animals and other organisms
☒	☐	Clinical data
☒	☐	Dual use research of concern
☒	☐	Plants

Methods

n/a	Involved in the study	
☒	☐	ChIP-seq
☒	☐	Flow cytometry
☒	☐	MRI-based neuroimaging

Antibodies

Antibodies used

Anti-green fluorescent protein (GFP) (1:2000), (NB100-1770S, Novus Biologicals)
Anti CD68 (1:100) (ab31630, Abcam, Cambridge, UK)
Anti PRRX1 (1:50) (HPA063566, Sigma)
Anti-type I collagen (COL1) (1:100) (H3884, Sigma)
Anti smooth muscle actin (aSMA) (1:1000) (A-2547, Sigma Aldrich)
Anti caveolin (1:100) (3238S, Cell Signaling Technology)
Alexa Fluor®488 (1:200) (A11029; Invitrogen, Thermo Fisher)
Alexa Fluor®568 (1:200) (A11036; Invitrogen, Thermo Fisher)
DAPI (1:10) (H-1200, Vectashield®, Vector Laboratories, Burlingame, CA, USA)

Validation

Anti-green fluorescent protein (GFP) was validated in spleen of commercial transgenic Sprague-Dawley rats containing the fluorescent protein GFP under the control of ubiquitin-C promoter (SD-TgGFP). Positive controls of the others antibodies used in this paper were validated by manufactures and previous studies of our group.
Negative controls were performed using the same protocol without the addition of the primary antibody

Animals and other research organisms

Policy information about studies involving animals; ARRIVE guidelines recommended for reporting animal research, and Sex and Gender in Research

Laboratory animals

The following commercial transgenic Sprague-Dawley rats containing the fluorescent protein GFP under the control of ubiquitin-C promoter (SD-TgGFP) and Hsd:Sprague Dawley® SD® from ENVIGO (https://www.inotivco.com/model/hsd-sprague-dawley-sd) were used in these studies

Wild animals

This study did not include any wild animal

Reporting on sex

Only females rats were used in these studies for two main reasons, the easy animal housing in the facilities and because they have a constant size and weight once they are adults. Besides, for future analyses of bone regeneration in osteoporosis models, these animals would be used as controls.

Field-collected samples

This study did not involved sample collection in the field

Ethics oversight

All experiments involving animals were approved by the Ethics Committee for Animal Experimentation of the University of Navarra (Comité de Ética para la Experimentación Animal, CEEA) and Navarra regional Government, CEEA# 105-17 and CEEA# 073-20.

Note that full information on the approval of the study protocol must also be provided in the manuscript.
